# Six New 3,5-Dimethylcoumarins from *Chelonopsis praecox*, *Chelonopsis odontochila* and *Chelonopsis pseudobracteata*

**DOI:** 10.1007/s13659-021-00318-9

**Published:** 2021-09-16

**Authors:** Chang-An Geng, Zhen-Tao Deng, Qian Huang, Chun-Lei Xiang, Ji-Jun Chen

**Affiliations:** 1 State Key Laboratory of Phytochemistry and Plant Resources in West China, Yunnan Key Laboratory of Natural Medicinal Chemistry, Kunming Institute of Botany, Chinese Academy of Sciences, Kunming, 650201 People’s Republic of China; 2 Key Laboratory for Plant Diversity and Biogeography of East Asia, Kunming Institute of Botany, Chinese Academy of Sciences, Kunming, 650201 People’s Republic of China; 3 University of Chinese Academy of Sciences, Beijing, 100049 People’s Republic of China

**Keywords:** 3,5-Dimethylcoumarins, 3-Methylcoumarin, *Chelonopsis*, Enzyme inhibition

## Abstract

Ten 3,5-dimethylcoumarins (**1**–**6** and **8**‒**11**) involving six new ones (**1**–**6**), together with a known 3-methylcoumarin (**7**), were isolated from the aerial parts of three *Chelonopsis* plants*, C. praecox*, *C. odontochila*, and *C. pseudobracteata*. The structures of the new compounds were determined by extensive HRESIMS, 1D and 2D NMR spectroscopic analyses. According to the substitution at C-5, these coumarins were classified into 5-methyl, 5-hydroxymethyl, 5-formyl, and 5-*nor* types. All the isolates were assayed for their inhibition on *α*-glucosidase, protein tyrosine phosphatase 1B, and T-cell protein tyrosine phosphatase in vitro.

**Supplementary Information:**

The online version contains supplementary material available at 10.1007/s13659-021-00318-9.

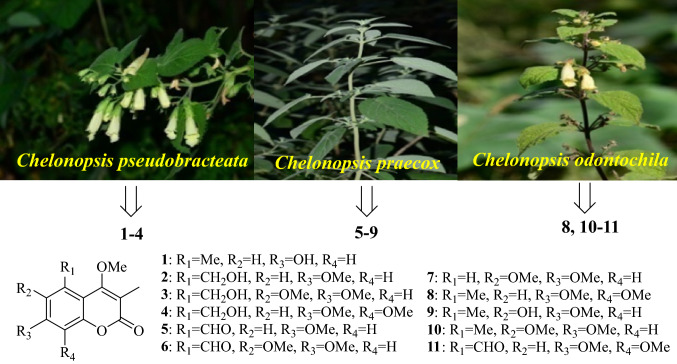

## Introduction

**Fig. 1:**
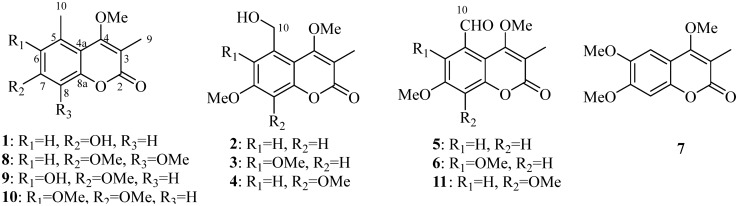
Chemical structures of compounds **1**–**11**

Coumarins with the benzo-*α*-pyrone core are widely distributed in plant kingdom and show a wide range of biological activities, including antimicrobial, antiviral, antidiabetic antiinflammatory, and antihypertensive activities, *etc*. [1]. Structurally, coumarins can be divided into simple coumarins, C-substituted coumarins, miscellaneous coumarins, biscoumarins, and triscoumarins. Besides hydroxy and methoxy groups, isopentenyl related C_5_-groups are the most common substituents present in coumarins, which are generally located at C-3, C-6, or C-8 positions by C–C linkage [2–4]. The methyl substituent in coumarin is very unusual, and only limited coumarins with the methylation at C-3, C-5, or C-6 positions have been reported. Currently, tens of 3,5-dimethylcoumarins have been isolated from *Clutia lanceolata* [5], *Clutia abyssinica* [6], *Juniperus sabina* [7], *Leucas inflata* [8], and *Sideritis pullulans* [9], but never from *Chelonopsis* plants. Our previous investigation on *Chelonopsis* plants yielded a series of diterpenoids with *α*-glucosidase inhibitory activity, *i.e*., ten *ent*-kauranes from *C. praecox* [10], and 13 *ent*-labdanes and 11 *ent*-kauranes from *C. odontochila* [11]. As a continuous search for antidiabetic candidates from natural sources [12–16], ten 3,5-dimethylcoumarins (**1**–**6** and **8**‒**11**) involving six new ones and one known 3-methylcoumarin (**7**) were first isolated from three *Chelonopsis* plants (Fig. 1). Herein, we report their isolation, structural elucidation, and enzymatic inhibition on *α*-glucosidase, protein tyrosine phosphatase 1B (PTP1B), and T-cell protein tyrosine phosphatase (TCPTP).

## Results and Discussion

### Structural Elucidation

Compound **1** had a chemical composition of C_12_H_12_O_4_ deduced by the [M + H]^+^ ion at *m/z* 221.0809, accounting for seven indices of hydrogen deficiency. The UV spectrum showed characteristic absorption at *λ*_max_ 321 nm for coumarins. The IR absorptions at 3204, 1681, 1610, and 1454 cm^−1^ were indicative for the presence of hydroxyl, carbonyl, and aromatic functionalities. In the ^1^H NMR spectrum, two *meta*-coupled aromatic protons at *δ*_H_ 6.93 (*J* = 2.4 Hz) and 6.87 (*J* = 2.4 Hz), one methoxy at *δ*_H_ 3.64, and two singlet methyls at *δ*_H_ 2.56 and 2.10 were well recognized (Table 1). The ^13^C NMR spectrum displayed 12 carbons comprising one carbonyl carbon, eight olefinic carbons, one methoxy, and two methyls. The ^1^H and ^13^C NMR data of **1** showed high resemblance with 6-hydroxy-3,5-dimethyl-4,7-dimethoxycoumarin (**9**) [5] except for the absence of a methoxy and an oxygenated methine in **9** being changed to be a methine in **1**. In the HMBC spectrum, the correlations from the methyl (*δ*_H_ 2.10) to C-2 (*δ*_C_ 164.0) and C-4 (*δ*_C_ 166.9), and from the methoxy (*δ*_H_ 3.64) to C-4 (*δ*_C_ 166.9) affirmed the 3-methyl and 4-methoxy substitution. Taking the ROESY correlations of Me-3/OMe-4/Me-5/H-6 into consideration, this compound was characterized to be 7-hydroxy-4-methoxy-3,5-dimethylcoumarin (**1**).

**Table 1:** ^1^H NMR data of compounds **1**–**7** (*δ* in ppm, *J* in Hz)

No.	**1**	**2**	**3**	**4**	**5**	**6**	**7**
5	−	−	−	−	−	−	7.07, s
6	6.87, d (2.4)	6.96, d (3.0)	−	6.99, s	7.24, d (2.7)	−	−
7	−	−	−	−	−	−	−
8	6.93, d (2.4)	6.77, d (3.0)	6.84, s	−	6.99, d (2.7)	6.89, s	6.85, s
3-Me	2.10, s	2.16, s	2.17, s	2.18, s	2.19, s	2.15, s	2.17, s
4-MeO	3.64, s	3.98, s	4.01, s	3.98, s	3.91, s	3.81, s	4.01, s
5-Me	2.56, s	−	−		−	−	−
5-HOCH_2_	−	4.89, s	4.99, s	4.89, s	−	−	−
5-OHC	−	−	−	−	10.74, s	10.44, s	−
6-HO	−	−	−	−	−	−	−
6-MeO	−	−	3.86, s	−	−	3,82, s	3.95, s
7-MeO	−	3.86, s	3.93, s	3.97, s	3.89, s	3.93, s	3.94, s
8-MeO	−	−	−	3.95, s	−	−	−

Compound **1** was measured in pyridine-*d*_5_, and **2**–**7** were measured in CDCl_3_

**Fig. 2:**
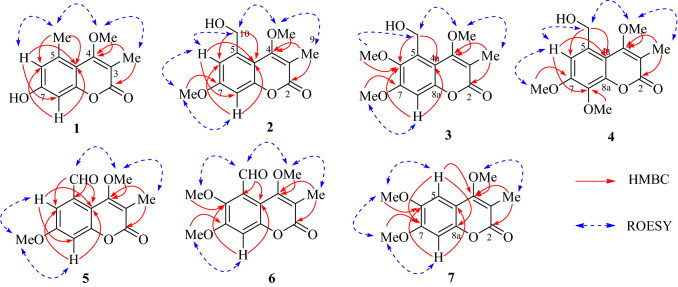
Selected 2D NMR correlations of compounds **1**–**7**

The molecular formula of **2** was assigned to be C_13_H_14_O_5_ by the positive HRESIMS ion at *m/z* 251.0897 ([M + H]^+^, calcd. for 251.0914). By comparing its ^1^H and ^13^C NMR data with those of **1**, the 5-methyl in **1** was changed to be a hydroxymethyl (*δ*_H_ 4.89, *δ*_C_ 56.2) in **2**, as well as an additional methoxyl group. The substitution of 5-hydroxymethyl and 7-methoxyl was confirmed by the HMBC correlations from H-10 (*δ*_H_ 4.89) to C-6 and C-4a, and from OMe-7 (*δ*_H_ 3.86) to C-7, as well as the ROESY correlations of H_3_-9/OMe-4/H_2_-10/H-6/OMe-7/H-8 (Fig. 2). Hence, compound **2** was defined as 5-hydroxymethyl-4,7-dimethoxy-3-methylcoumarin.

Compounds **3** and **4** were a pair of isomers with the same molecular formula of C_14_H_16_O_6_, indicating an additional CH_2_O moiety than **2**. In their ^1^H and ^13^C NMR spectra, three methoxy groups (*δ*_H_ 3.86, 3.93, 4.01 and *δ*_C_ 56.2, 61.9, 62.1 for **3**; *δ*_H_ 3.95, 3.97, 3.98 and *δ*_C_ 56.5, 61.67, 61.70 for **4**) were obviously recognized (Table 2), suggesting compounds **3** and **4** should be the methoxylated derivatives of **2**. The position of the additional methoxy in **3** and **4** were unambiguously determined by analyzing their ROESY experiments. In the ROESY spectrum of **3**, the correlation peaks of H_3_-9/OMe-4/H_2_-10/OMe-6 and OMe-7/H-8 revealed the methoxy at C-6 position. Similarly, the ROESY signals of H_3_-9/OMe-4/H_2_-10/H-6/OMe-7 in **4** supported the methoxy at C-8 position. Thus, compounds **3** and **4** were concluded as 5-hydroxymethyl-4,6,7-trimethoxy-3-methylcoumarin (**3**) and 5-hydroxymethyl-4,7,8-trimethoxy-3-methylcoumarin (**4**), respectively.

**Table 2:** ^13^C NMR data of compounds **1**–**7** (*δ* in ppm, *J* in Hz)

No.	**1**	**2**	**3**	**4**	**5**	**6**	**7**
2	164.0, C	164.4, C	164.1, C	163.7, C	163.6, C	164.1, C	165.2, C
3	108.9, C	110.4, C	111.5, C	111.2, C	111.9, C	110.0, C	109.6, C
4	166.9, C	165.6, C	165.3, C	165.2, C	164.5, C	163.7, C	164.2, C
4a	108.8, C	108.5, C	108.6, C	109.6, C	110.1, C	108.3, C	109.9, C
5	137.6, C	139.7, C	130.5, C	133.2, C	135.5, C	130.0, C	103.5, CH
6	117.2, CH	113.8, CH	145.0, C	110.0, CH	111.5, CH	143.2, C	146.5, C
7	161.2, C	161.9, C	155.6, C	154.2, C	161.5, C	156.0, C	152.5, C
8	101.3, CH	100.3, CH	100.5, CH	135.9, C	105.6, CH	101.5, CH	100.0, CH
8a	156.0, C	155.4, C	151.0, C	147.8, C	154.2, C	149.5, C	148.2, C
3-Me	10.6, CH_3_	11.2, CH_3_	11.0, CH_3_	11.3, CH_3_	10.9, CH_3_	10.9, CH_3_	10.9, CH_3_
4-MeO	60.3, CH_3_	61.6, CH_3_	61.9, CH_3_	61.7, CH_3_	60.9, CH_3_	60.7, CH_3_	61.4, CH_3_
5-Me	22.1, CH_3_	−	−	−	−	−	−
5-HOCH_2_	−	64.6, CH_2_	56.2, CH_2_	64.9, CH_2_	−	−	−
5-OHC	−	−	−	−	192.3, CH	192.4, CH	−
6-OMe	−	−	62.1, CH_3_	−	−	62.7, CH_3_	56.6, CH_3_
7-OMe	−	55.9, CH_3_	56.2, CH_3_	56.5, CH_3_	56.1, CH_3_	56.4, CH_3_	56.6, CH_3_
8-OMe	−	−	−	61.7, CH_3_	−	−	−

Compound **1** was measured in pyridine-*d*_5_, and **2**–**7** were measured in CDCl_3_

Compound **5** had a chemical composition of C_13_H_12_O_5_ according to the protonated ion at *m/z* 249.0744 in the HRESIMS data. In the ^1^H NMR spectrum, the presence of a formyl group at *δ*_H_ 10.74, two *meta*-coupled aromatic protons at *δ*_H_ 7.24 (*J* = 2.7 Hz) and 6.99 (*J* = 2.7 Hz), two methoxys at *δ*_H_ 3.91 and 3.89, and a methyl at *δ*_H_ 2.19 were easily recognized. By comparing with **2**, compound **5** had an additional formyl group at *δ*_H_ 10.74 and *δ*_C_ 192.3, but with the absence of a hydroxymethyl group (*δ*_H_ 4.89 and *δ*_C_ 64.6), indicating the dehydrogenated derivative of **2**. The formyl group was assigned at C-5 by the ROESY correlations of H_3_-9/OMe-4/H-10, and HMBC correlation from H-6 to C-10 and from H-10 to C-5, C-6 and C-4a. Consequently, compound **5** was defined as 5-formyl-4,7-dimethoxy-3-methylcoumarin.

The molecular formula of **6** was assigned as C_14_H_14_O_6_ by the [M + H]^+^ ion at *m/z* 279.0906 in positive HRESIMS spectrum. In the ^1^H NMR spectrum, one formal at *δ*_H_ 10.44, one aromatic singlet at *δ*_H_ 6.89, three methoxy groups at *δ*_H_ 3.81, 3.82, and 3.93, and one methyl group at *δ*_H_ 2.15 were observed, showing an extra methoxy than **5**. The above deduction was consistent with that two *meta*-coupled protons at *δ*_H_ 7.24 and 6.99 in **5** was changed to be an aromatic singlet at *δ*_H_ 6.89 in **6**. By analyzing the ROESY experiment, the correlations of H_3_-9/OMe-4/H-10/OMe-6 and OMe-7/H-8 demonstrated the structure of 5-formyl-4,6,7-trimethoxy-3-methylcoumarin (**6**).

Compound **7** was assigned with the chemical formula of C_13_H_14_O_5_ by the [M + H]^+^ ion at *m/z* 251.0898 in positive HRESIMS spectrum. In the ^1^H NMR spectrum, two aromatic protons at *δ*_H_ 7.07 and 6.85, three methoxy groups at *δ*_H_ 4.01, 3.95, and 3.94, and a methyl group at *δ*_H_ 2.17, were recognized. Compared with 4,6,7-trimethoxy-3,5-dimethylcoumarin (**10**) [6], the 5-methyl in **10** was absent in **7** but with an extra aromatic singlet at *δ*_H_ 7.07. This proton (*δ*_H_ 7.07) was assigned to be H-5 by the HMBC correlation from H-5 to C-4, and ROESY correlations of H-5/OMe-6 and H-8/OMe-7. Thus, compound **7** was deduced as 4,6,7-trimethoxy-3-methylcoumarin, the demethylated derivative of **10**. Although this compound has been synthesized by methylation of 4-hydroxy-6,7-dimethoxy-3-methylcoumarin in 1949 [17], it is the first report of its natural occurrence and NMR spectroscopic data.

The known coumarins were determined to be 4,7,8-trimethoxy-3,5-dimethylcoumarin (**8**) [7], 6-hydroxy-4,7-dimethoxy-3,5-dimethylcoumarin (**9**) [5], 4,6,7-trimethoxy-3,5-dimethylcoumarin (**10**) [6], and 5-formyl-4,7,8-trimethoxy-3-methylcoumarin (**11**) [8] by comparing their ^1^H and ^13^C NMR data with those in the literatures.

In order to evaluate their antidiabetic potency, all the coumarins were assayed for their inhibitory activity on *α*-glucosidase, PTP1B, and TCPTP. As shown in Table 3, all the compounds showed only weak or no inhibition to three enzymes at the concentration of 200 μM. According to the previous study [5], this type of coumarins could enhance the glucose-triggered secretion of insulin from murine islets. Thus, further studies will be needed to reveal their targets and mechanisms in exerting hypoglycemic effects.

**Table 3:** Inhibitory rates of the isolates (200 μM) on *α*-glucosidase, PTP1B, and TCPTP

No.	Inhibition rates (%)		
	*α*-glucosidase	PTP1B	TCPTP
**1**	11.1 ± 4.1	26.2 ± 2.9	5.0 ± 1.3
**2**	22.3 ± 6.9	9.8 ± 2.5	‒ 4.9 ± 2.1
**3**	6.2 ± 4.6	6.8 ± 3.4	‒ 9.3 ± 3.2
**4**	26.4 ± 9.9	13.4 ± 5.9	‒ 9.1 ± 1.5
**5**	10.9 ± 9.1	19.0 ± 4.6	1.7 ± 0.5
**6**	0.8 ± 1.0	8.7 ± 3.5	‒ 3.2 ± 1.1
**7**	12.0 ± 3.7	13.6 ± 3.9	25.9 ± 2.4
**8**	‒ 4.5 ± 0.9	27.8 ± 7.9	7.1 ± 3.8
**9**	12.5 ± 3.9	9.5 ± 2.2	27.8 ± 4.1
**10**	11.2 ± 3.7	25.1 ± 1.9	‒ 3.1 ± 0.6
**11**	9.5 ± 2.3	35.7 ± 2.4	‒ 5.3 ± 2.0
Acarbose	87.9 ± 0.4	‒	‒
Na_3_VO_4_	‒	69.7 ± 5.2	45.4 ± 2.1

Data were expressed as means ± SD (n = 3) from three independent experiments

## Experimental Section

### General Experimental Procedures

A Jasco model 1020 digital polarimeter (Jasco Corp., Tokyo, Japan) was used to measure optical rotations. UV and IR data were obtained using a Shimadzu UV2401PC spectrophotometer (Shimadzu, Kyoto, Japan) and a Nicolet iS10 spectrometer (Thermo Fisher Scientific, Madison, WI, USA), respectively. A Waters AutoSpec Premier P776 mass spectrometer (Waters, Milford, MA, USA) or a Shimadzu LCMS-IT-TOF mass spectrometer (Shimadzu, Kyoto, Japan) was used to acquire the high-resolution mass spectra. ECD spectra were recorded on an Applied Photophysics Chirascan apparatus (Applied Photophysics, Surrey, UK). NMR spectra were obtained by using DRX-500, Avance III-600, and Ascend™ 800 MHz spectrometers (Bruker, Karlsruhe, Germany). TLC detection was run on silica gel plates (60 F254). Silica gel (200–300 mesh, Qingdao Makall group Co. Ltd., Qingdao, China) and Sephadex LH-20 (GE Healthcare Bio-Sciences AB, Uppsala, Sweden) were used for column chromatography. A Dr-Flash II apparatus was applied to accomplish the MPLC separations. HPLC purifications were conducted on a Shimadzu LC-CBM-20 system (Shimadzu, Kyoto, Japan), equipped with an Agilent Eclipse XDB-C_18_ column (5 μm, 9.4 × 250 mm).

### Plant Materials

The aerial parts of three *Chelonopsis* plants were collected in October 2016 from Lijiang, Yunnan Province of China, which were authenticated to be *Chelonopsis odontochila* Diels, *Chelonopsis pseudobracteata* C. Y. Wu et H. W. Li, and *Chelonopsis praecox* Weckerle and F. Huber by Dr. Chun-Lei Xiang. Voucher specimens (Nos. 2016102101, 2016102102, 2016102103) were deposited in the Laboratory of Anti-virus and Natural Medicinal Chemistry, Kunming Institute of Botany, Chinese Academy of Sciences, China.

### Extraction and Isolation

The air-dried plants of *C. pseudobracteata* (6 kg) were powdered and extracted three times with 90% aqueous EtOH (25 L × 3) at room temperature. The extract was evaporated under reduced pressure, and the residue was suspended in H_2_O and partitioned with CHCl_3_. The CHCl_3_ extraction (95 g) was subjected to silica gel column chromatography (Si CC) and eluted with an acetone-petroleum ether solvent system (from 10:90 to 50:50, *v/v*) to afford seven fractions (A–G). Fraction C (19.8 g) was subjected over MCI gel CHP 20P column (H_2_O–MeOH, 50:50–0:100) to provide five fractions, Frs. C1–C5. Fr. C_3_ (3.4 g) was purified via Si CC (EtOAc-petroleum ether, 10:90–50:50) to give three fractions, Frs. C3-1–C3-3. Fr. C3-1 (600 mg) was purified by Sephadex LH-20 CC (MeOH–CHCl_3_, 50:50) and semi-preparative HPLC (H_2_O–MeCN, 36:64) to give compounds **1** (16 mg) and **3** (18 mg). Compounds **2** (25 mg) and **4** (38 mg) were obtained from Fr. C3-2 (750 mg) by Sephadex LH-20 CC (MeOH–CHCl_3_, 50:50) and semi-preparative HPLC (H_2_O–MeCN, 50:50).

The air-dried plants of *C. praecox* (25 kg) were powdered and extracted three times with 90% aqueous EtOH (100 L × 3) at room temperature. The combined EtOH extract was concentrated and partitioned between H_2_O and CHCl_3_. The CHCl_3_ extraction (380 g) was subjected to Si CC (4.0 kg, 30 × 100 cm), using a gradient elution of EtOAc-petroleum ether (from 10:90 to 100:0) to afford seven fractions (A–G). MPLC separation of Fr. D (46 g) by using a CHP20P MCI gel column (H_2_O-MeOH, from 50:50 to 0:100) provided five fractions, Frs. D1–D5. Fr. D3 (1.3 g) was separated by Si CC (EtOAc-CHCl_3_, 2:98–50:50) to afford five fractions, Frs. D3-1–D3-5. Fr. D3-1 (265 mg) was purified by Si CC (acetone-petroleum ether, 5:95), and semi-preparative HPLC (H_2_O–MeCN, 42:58) to yield compounds **8** (25 mg), **5** (18 mg), and **6** (18 mg). Fr. E (17 g) was subjected to MPLC to give five fractions, Frs. E1–E5. Compounds **9** (5 mg) and **7** (25 mg) were obtained from Fr. E3 after repeated Si CC (acetone-petroleum ether, 10:90) and Sephadex LH-20 (MeOH–CHCl_3_, 50:50), and semi-preparative HPLC (MeCN–H_2_O, 40:60).

Air-dried and powdered plants of *C. odontochila* (8.0 kg) were extracted with 90% aqueous EtOH (35 L × 3) at room temperature. The combined EtOH extract was concentrated and partitioned between H_2_O and CHCl_3_. The CHCl_3_ extract (160 g) was chromatographed on a silica gel column (1.3 kg, 30 × 100 cm), and eluted with acetone-petroleum ether gradient (from 0:100 to 100:0) to yielded seven fractions, Frs. A–G. MPLC separation of Fr. C (15 g) with MCI gel CHP 20P column (H_2_O–MeOH, from 50:50 to 0:100) gave rise to five fractions, Frs. C1–C5. Fraction C3 (2.8 g) was chromatographed on a silica gel column (acetone-CHCl_3_, from 10:90 to 100:0) to provide four fractions, Frs. C3-1–C3-4. Compound **11** (12 mg) was purified from Fr. C3-2 (500 mg) by Sephadex LH-20 CC (MeOH–CHCl_3_, 50:50), and semi-preparative HPLC (H_2_O–MeCN, 64:36). After repeated separation over Si CC (acetone-petroleum ether, 15:85), Sephadex LH-20 CC (MeOH–CHCl_3_, 50:50), and semi-preparative HPLC (H_2_O–MeCN, 60:40), compounds **10** (27 mg) and **8** (21 mg) were obtained from Fr. C3-3 (470 mg).

### Spectroscopic Data of Compounds

#### 7-Hydroxy-4-Methoxy-3,5-Dimethylcoumarin (**1**)

White amorphous powder; UV (MeOH) *λ*_max_ (log *ε*): 321 (3.99) nm; IR (KBr) *ν*_max_: 3204, 1681, 1610, 1567, 1454, 1378, 1358, 1344, 1257, 1154, 1102, 1074, 1016 cm^−1^; ^1^H and ^13^C NMR data, see Tables 1 and 2; positive HRESIMS *m/z* 221.0809 [M + H]^+^ (calcd. for C_12_H_13_O_4_, 221.0808).

#### 5-Hydroxymethyl-4,7-Dimethoxy-3-Methylcoumarin (**2**)

White amorphous powder; UV (MeOH) *λ*_max_ (log *ε*): 222 (3.91), 320 (3.90) nm; IR (KBr) *ν*_max_: 3398, 1663, 1616, 1592, 1558, 1453, 1432, 1369, 1336, 1251, 1197, 1151, 1083, 1047, 1012, 946 cm^−1^; ^1^H and ^13^C NMR data, see Tables 1 and 2; positive HRESIMS *m/z* 251.0897 [M + H]^+^ (calcd. for C_13_H_15_O_5_, 251.0914).

#### 5-Hydroxymethyl-4,6,7-Trimethoxy-3-Methylcoumarin (**3**)

White amorphous powder; UV (MeOH) *λ*_max_ (log *ε*): 294 (3.75), 328 (4.00) nm; IR (KBr) *ν*_max_: 3434, 1683, 1603, 1562, 1452, 1418, 1366, 1331, 1262, 1224, 1161, 1130, 1081, 1061, 1004, 987 cm^−1^; ^1^H and ^13^C NMR data, see Tables 1 and 2; positive HRESIMS *m/z* 281.1039 [M + H]^+^ (calcd. for C_14_H_17_O_6_, 281.1020).

#### 5-Hydroxymethyl-4,7,8-Trimethoxy-3-Methylcoumarin (**4**)

White amorphous powder; UV (MeOH) *λ*_max_ (log *ε*): 318 (4.07) nm; IR (KBr) *ν*_max_: 3435, 1687, 1595, 1571, 1457, 1421, 1337, 1274, 1136, 1096, 1052 1012 cm^−1^; ^1^H and ^13^C NMR data, see Tables 1 and 2; positive HRESIMS *m/z* 281.1014 [M + H]^+^ (calcd. for C_14_H_17_O_6_, 281.1020).

#### 5-Formyl-4,7-Dimethoxy-3-Methylcoumarin (**5**)

White amorphous powder; UV (MeOH) *λ*_max_ (log *ε*): 218 (3.09), 291 (2.74), 328 (2.83) nm; IR (KBr) *ν*_max_: 3426, 1725, 1688, 1605, 1448, 1367, 1336, 1258, 1168, 1089, 953 cm^−1^; ^1^H and ^13^C NMR data, see Tables 1 and 2; positive HRESIMS *m/z* 249.0744 [M + H]^+^ (calcd. for C_13_H_13_O_5_, 249.0758).

#### 5-Formyl-4,6,7-Trimethoxy-3-Methylcoumarin (**6**)

White amorphous powder; UV (MeOH) *λ*_max_ (log *ε*): 207 (3.67), 224 (3.63), 290 (3.20), 329 (3.45) nm; IR (KBr) *ν*_max_: 3420, 1721, 1693, 1603, 1455, 1388, 1369, 1266, 1226, 1078, 1006, 959 cm^−1^; ^1^H and ^13^C NMR data, see Tables 1 and 2; positive HRESIMS *m/z* 279.0906 [M + H]^+^ (calcd. for C_14_H_15_O_6_, 279.0863).

#### 4,6,7-Trimethoxy-3-Methylcoumarin (**7**)

Colorless gum; UV (MeOH) *λ*_max_ (log *ε*): 208 (2.99), 222 (2.83), 287 (2.30), 333 (2.58) nm; IR (KBr) *ν*_max_: 3431, 1709, 1620, 1580, 1453, 1372, 1337, 1249, 1215, 1162, 1025, 994 cm^−1^; ^1^H and ^13^C NMR data, see Tables 1 and 2; positive HRESIMS *m/z* 251.0898 [M + H]^+^ (calcd. for C_13_H_15_O_5_, 251.0914).

### In Vitro Enzyme Inhibition Assays

In this study, three enzymes closely related to diabetes, namely *α*-glucosidase, PTP1B, and TCPTP, were applied to assess the antidiabetic potency of compounds. Enzyme inhibition was assayed in accordance with the previous reports [18, 19]. Acarbose (for *α*-glucosidase) and Na_3_VO_4_ (for PTP1B and TCPTP) were used as the positive controls.

## Supporting Information

1D and 2D NMR, HRMS, UV and IR spectra of compounds **1**−**7**.

## Supplementary Information

Below is the link to the electronic supplementary material.

Supplementary file1 (PDF 4181 kb)

## References

[1] Hussain MI, Syed QA, Khattak MNK, Hafez B, Rrigosa MJ, El-Keblawy A (2019). Biologia.

[2] Estbvez-Braun AG (1997). Nat. Prod. Rep..

[3] Murray RDH (1995). Nat. Prod. Rep..

[4] Murray RDH (1989). Nat. Prod. Rep..

[5] Ahmed S, Nur-e-Alam M, Parveen I, Coles SJ, Hafizur RM, Hameed A, Orton JB, Threadgill MD, Yousaf M, Alqahtani AM, Al-Rehaily AJ (2020). Phytochemistry.

[6] Waigh RD, Zerihun BM, Maitland DJ (1991). Phytochemistry.

[7] J.de Pascual, A. San Feliciano, J.M. Miguel del Corral, A.F. Barrero, M. Rubio, L. Muriel, Phytochemistry **20**, 2778–2779 (1981)

[8] M.H. Al Yousuf, A.K. Bashir, G. Blunden, M.-H. Yang, A.V. Patel, Phytochemistry **51**, 95–98 (1999)

[9] Faiella L, Dal Piaz F, Bader A, Braca A (2014). Phytochemistry.

[10] Deng ZT, Geng CA, Yang TH, Xiang CL, Chen JJ (2019). Fitoterapia.

[11] Deng ZT, Chen JJ, Geng CA (2020). Bioorg. Chem..

[12] He XF, Geng CA, Huang XY, Ma YB, Zhang XM, Chen JJ (2019). Nat. Prod. Bioprospect..

[13] He XF, Chen JJ, Li TZ, Hu J, Huang XY, Zhang XM, Guo YQ, Geng CA (2021). Chin. J. Chem..

[14] He XF, Chen JJ, Li TZ, Hu J, Zhang XM, Geng CA (2021). Bioorg. Chem..

[15] He XF, Wang HM, Geng CA, Hu J, Zhang XM, Guo YQ, Chen JJ (2020). Phytochemistry.

[16] Huang Q, Chen JJ, Pan Y, He XF, Wang Y, Zhang XM, Geng CA (2021). J. Pharm. Biomed. Anal..

[17] Jones GH, Mackenzie JBD, Robertson A, Whalley WB (1949). J. Chem. Soc..

[18] Yan DX, Geng CA, Yang TH, Huang XY, Li TZ, Gao Z, Ma YB, Peng H, Zhang XM, Chen JJ (2018). Fitoterapia.

[19] Zhang CC, Geng CA, Huang XY, Zhang XM, Chen JJ (2019). J. Agric. Food Chem..

