# Supplementary material for: Six New 3,5-Dimethylcoumarins from Chelonopsis praecox, Chelonopsis odontochila and Chelonopsis pseudobracteata
Source: Nat Prod Bioprospect. 2021 Sep 16;11(6):643–9. doi: 10.1007/s13659-021-00318-9 (PMC8599598; doi:10.1007/s13659-021-00318-9)
Supplement: Supplementary file 1 — Supplementary file1 (PDF 4181 kb) [file 13659_2021_318_MOESM1_ESM.pdf]

Supplementary Data

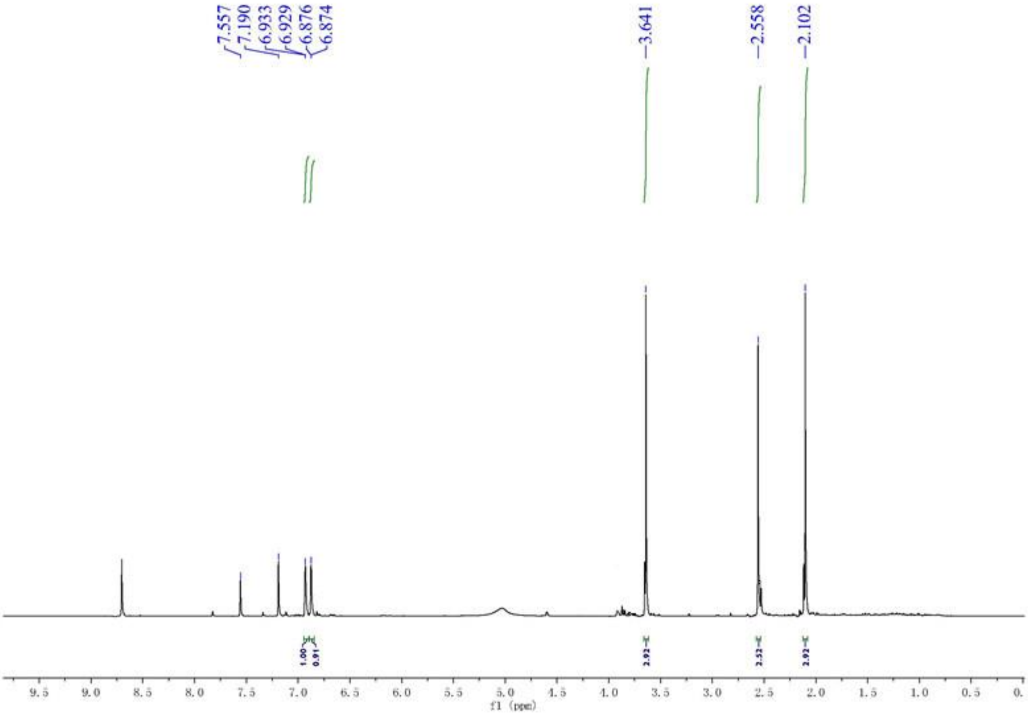

^{1}H-NMR spectrum of **1** recorded in pyridine-d_5 at 500MHz

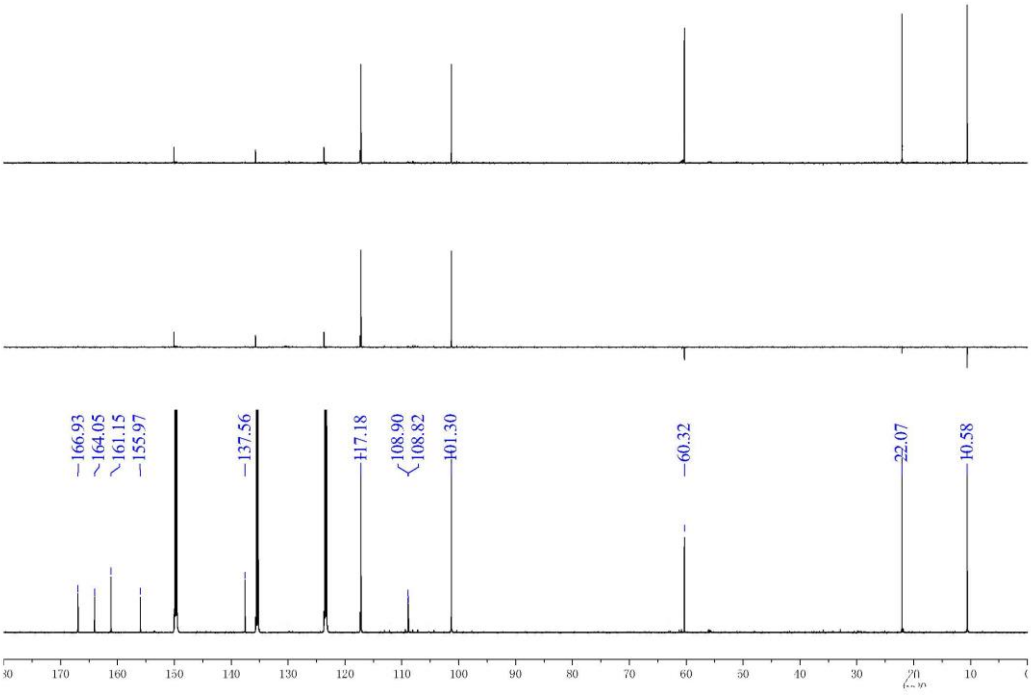

^{13}C-NMR spectrum of **1** recorded in pyridine-d_5 at 125MHz

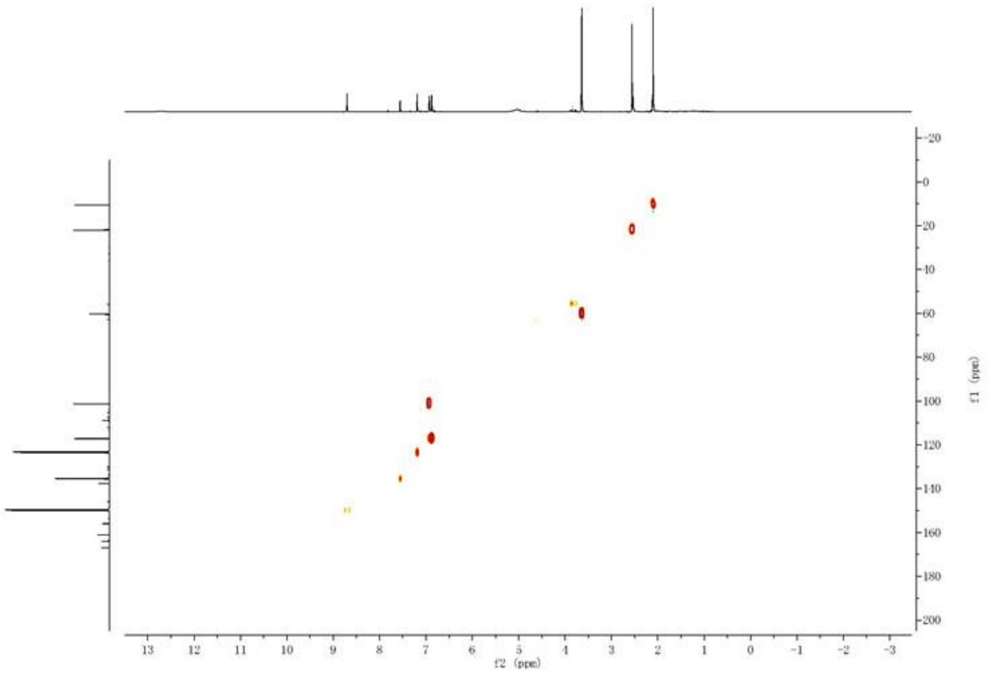

HSQC spectrum of **1** recorded in pyridine-d_5

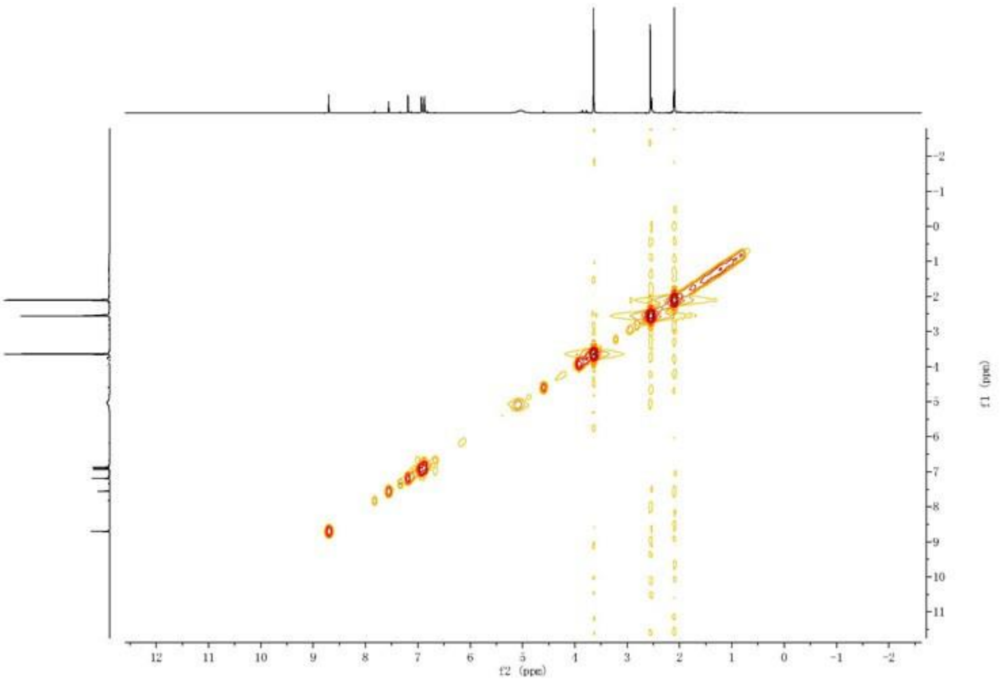

^1H-^1H COSY spectrum of **1** recorded in pyridine-d_5

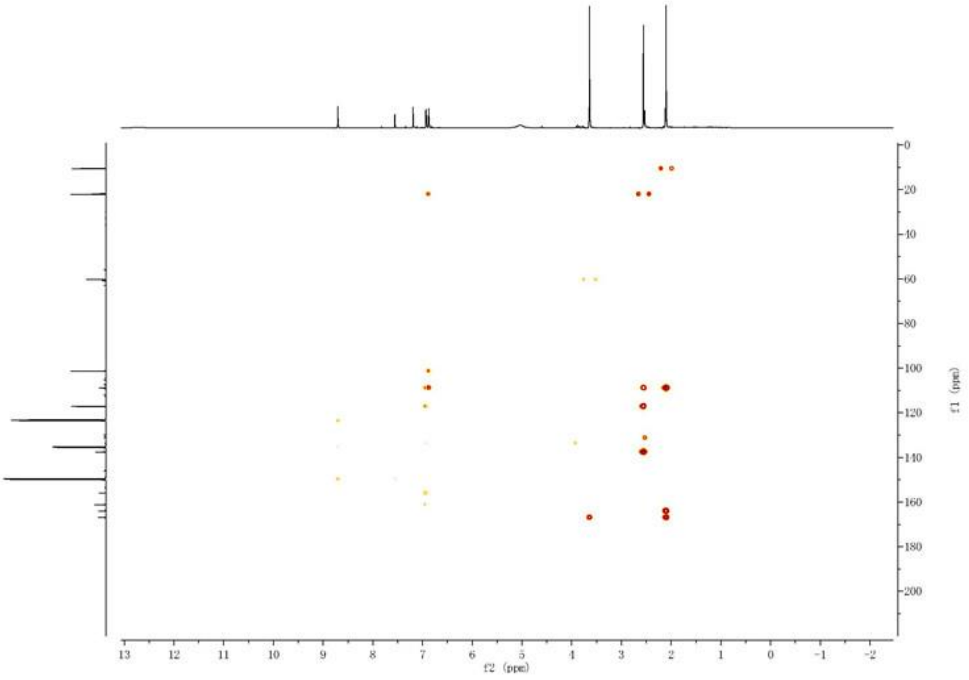

HMBC spectrum of **1** recorded in pyridine-d_5

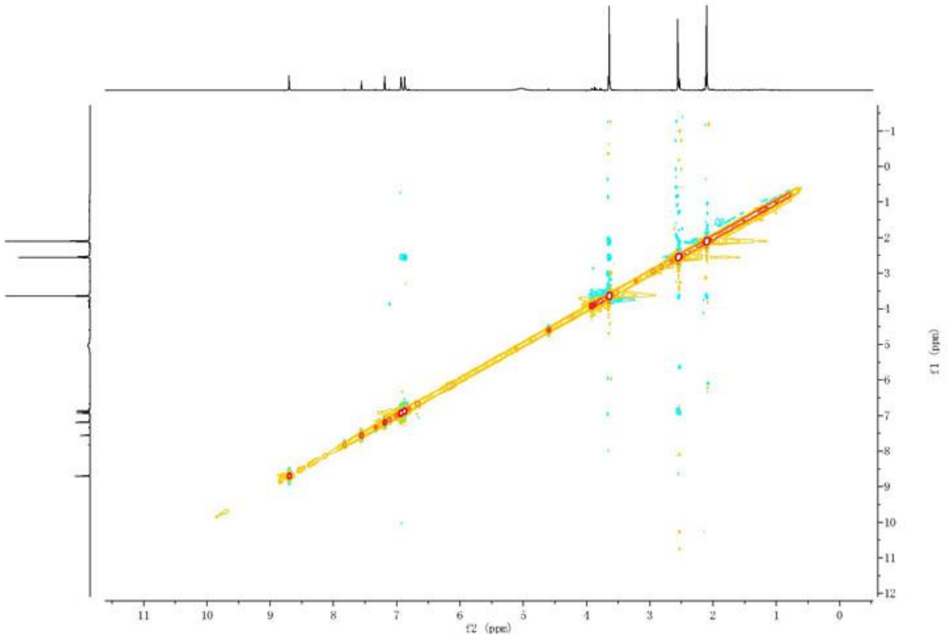

ROESY spectrum of **1** recorded in pyridine-d_5

Data File: E:\百度云同步盘\昆植所\实验室\LC-MS结果\假具苞铃子香\2018-01-31_2018131_Cp-A-4_10.lcd

Elmt	Val.	Min	Max	Elmt	Val.	Min	Max	Elmt	Val.	Min	Max	Use Adduct
H	1	0	300	O	2	0	50	Br	1	0	0	H
C	4	0	150	S	2	0	0					Na
N	3	0	0	Cl	1	0	0					

Error Margin (ppm):	50	DBE Range:	0.0 - 30.0	Electron Ions:	both
HC Ratio:	unlimited	Apply N Rule:	yes	Use MSn Info:	no
Max Isotopes:	all	Isotope RI (%):	1.00	Isotope Res:	10000
MSn Iso RI (%):	75.00	MSn Logic Mode:	AND	Max Results:	800

Event#: 1 MS(E+) Ret. Time : 10.573 Scan# : 2129

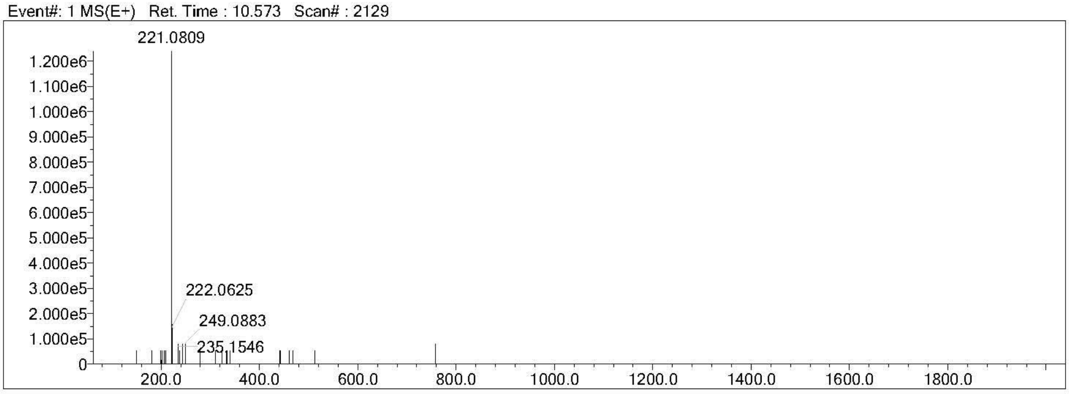

Measured region for 221.0809 m/z

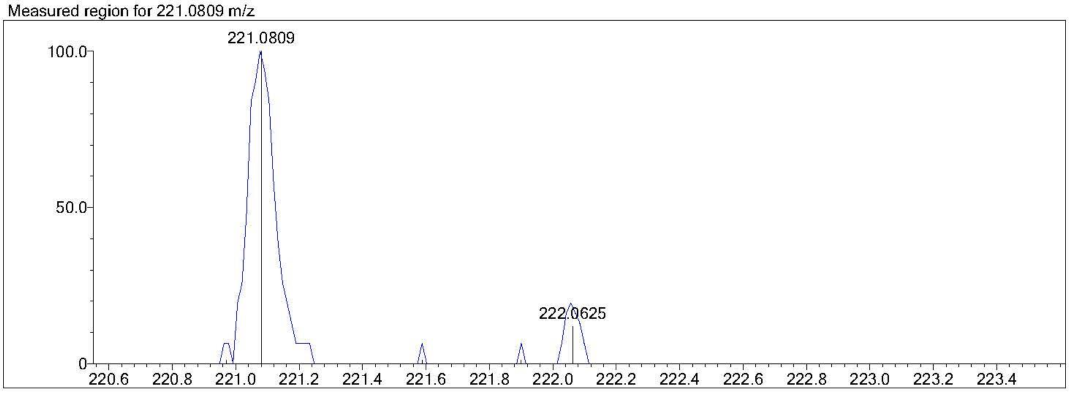

C12 H12 O4 [M+H]+ : Predicted region for 221.0808 m/z

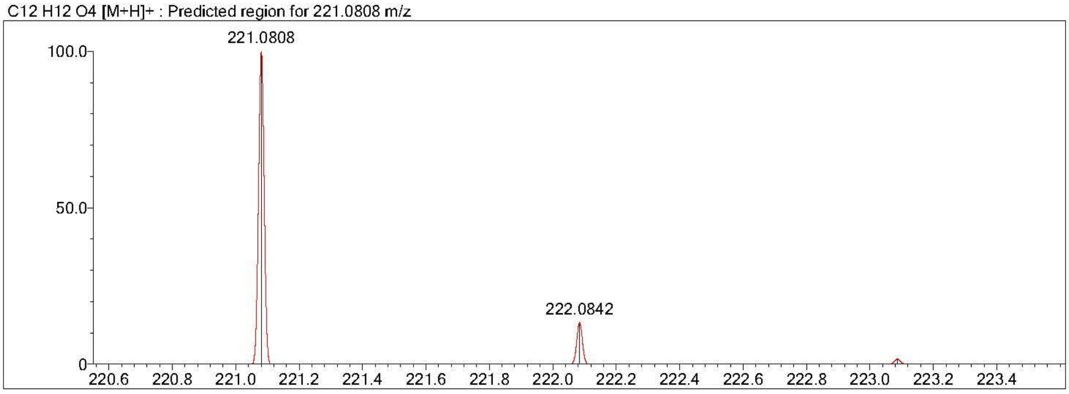

Rank	Score	Formula (M)	Ion	Meas. m/z	Pred. m/z	Df. (mDa)	Df. (ppm)	Iso	DBE
1	56.49	C12 H12 O4	[M+H]+	221.0809	221.0808	0.1	0.45	56.49	7.0

HR-ESI-MS spectrum of **1**

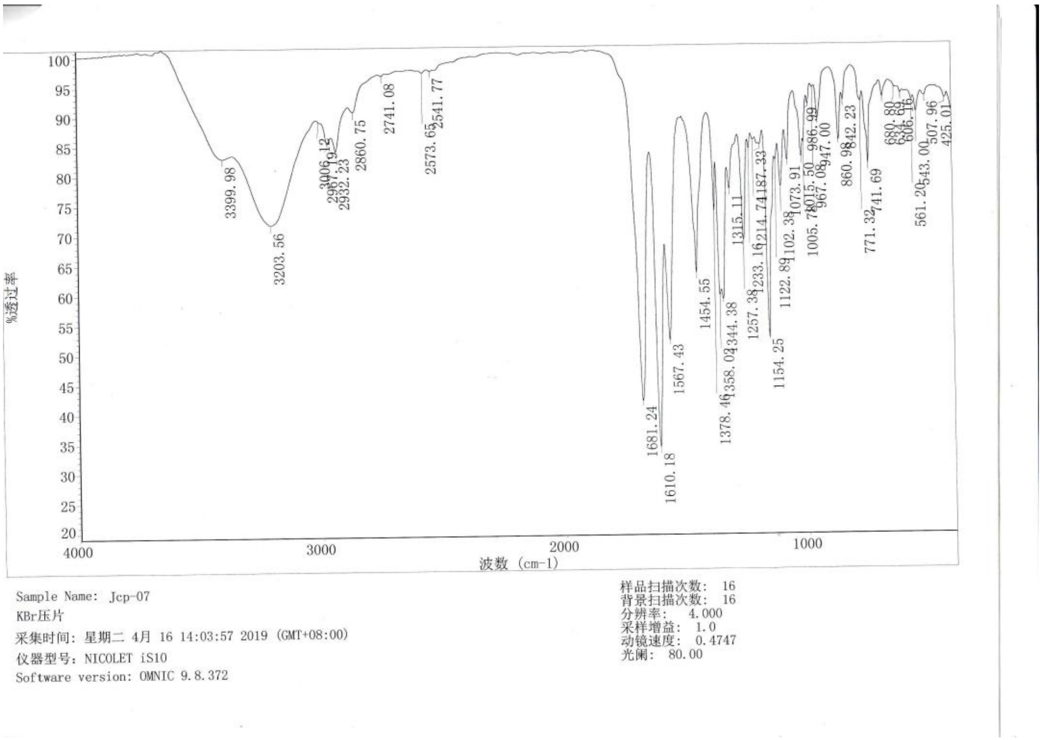

IR spectrum of **1**

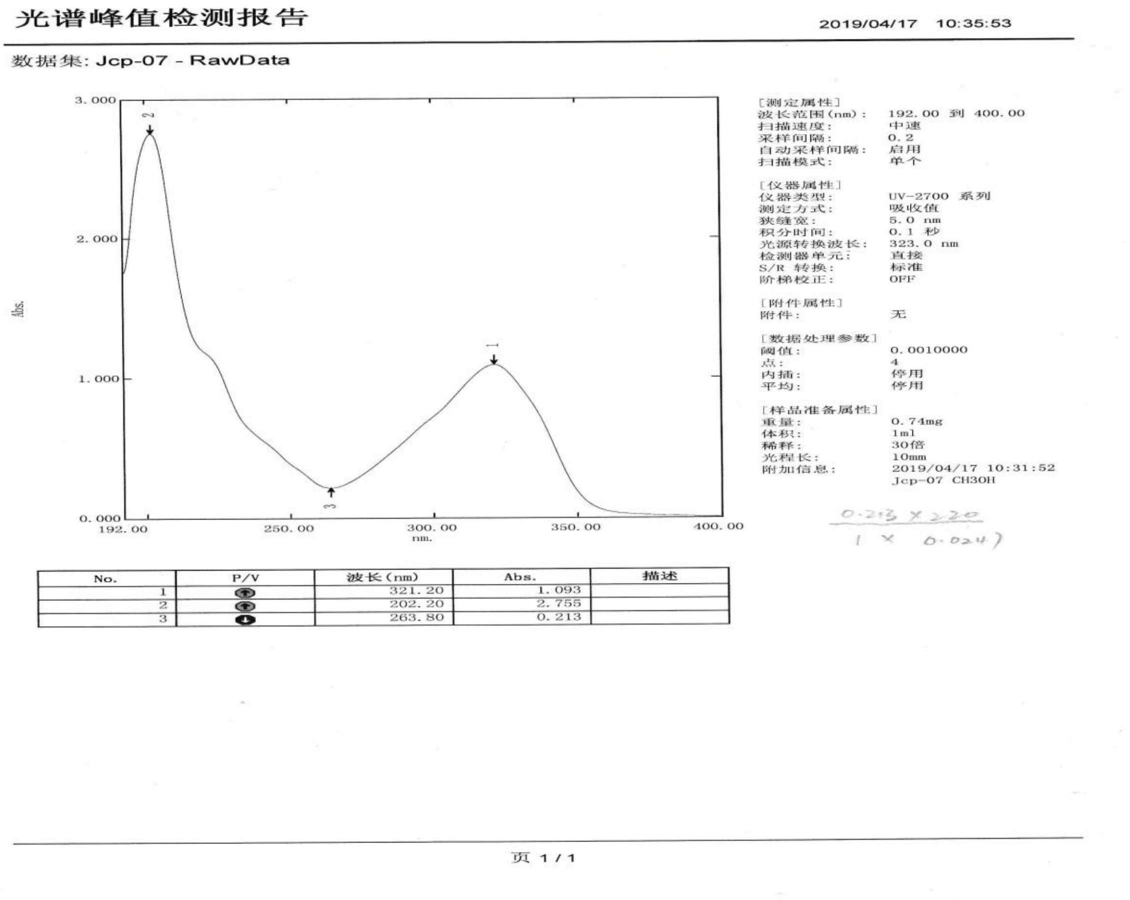

UV spectrum of **1**

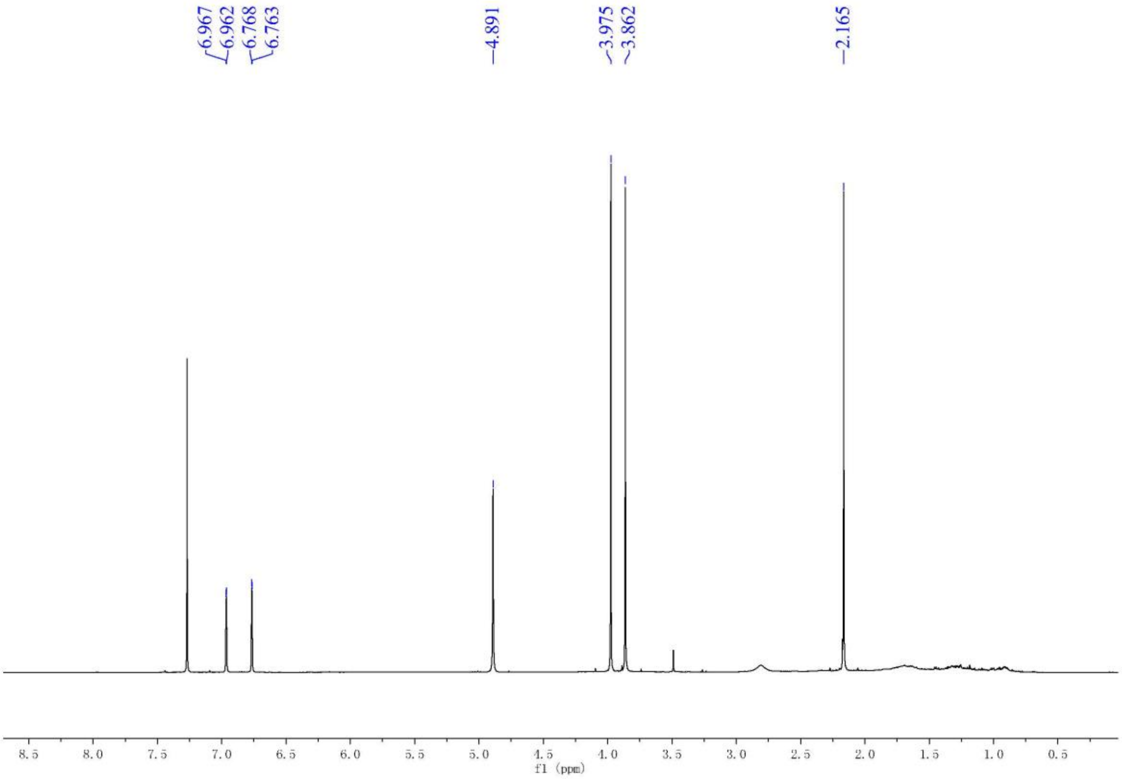

^{1}H-NMR spectrum of **2** recorded in $CDCl_3$ at 600MHz

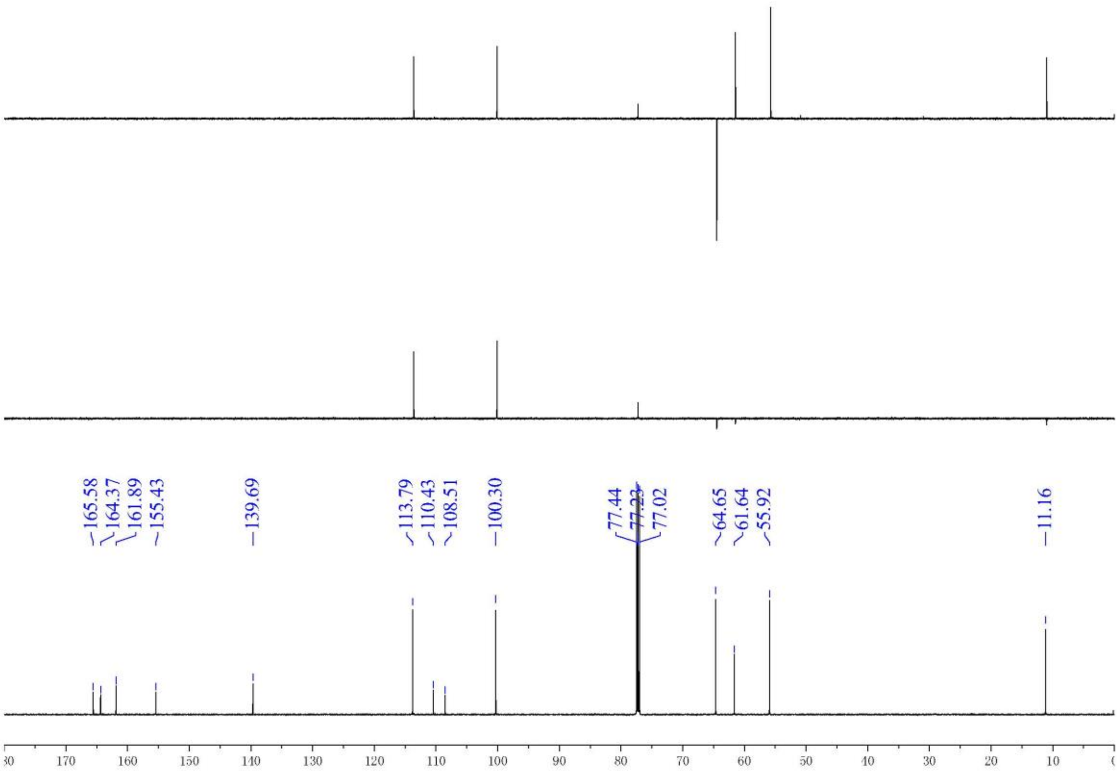

^{13}C-NMR spectrum of **2** recorded in $CDCl_3$ at 150MHz

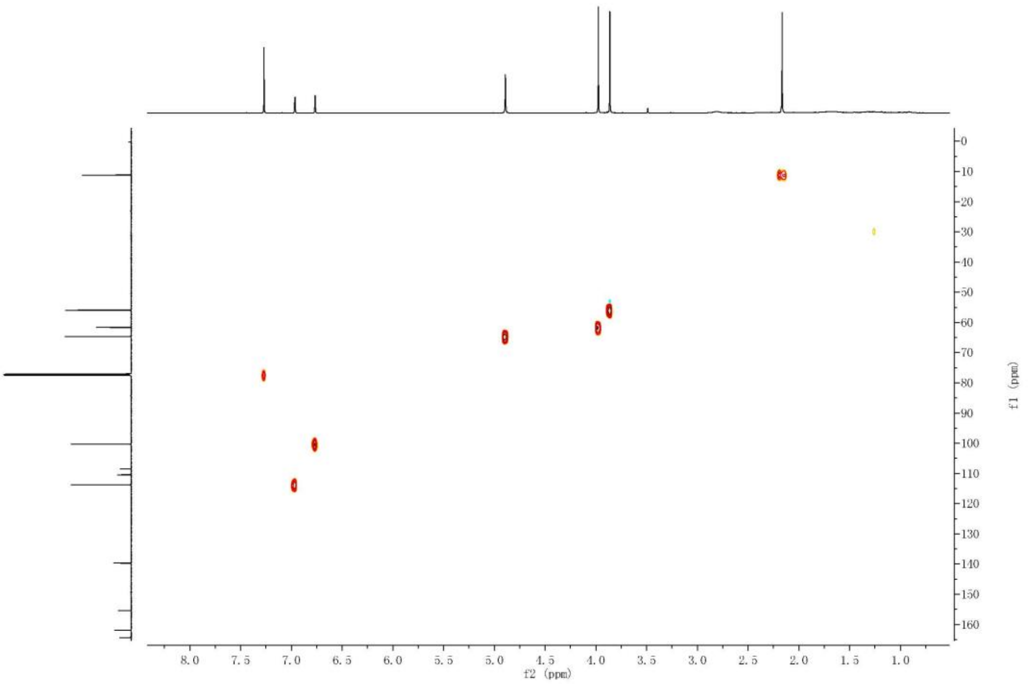

HSQC spectrum of **2** recorded in $CDCl_3$

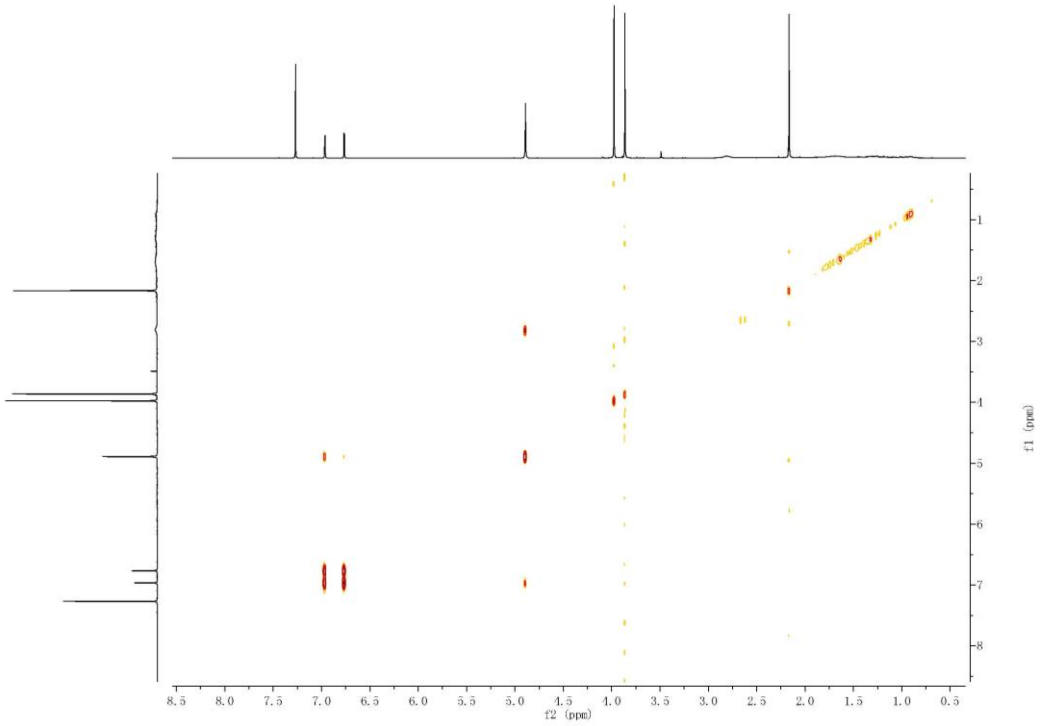

^{1}H-^{1}H COSY spectrum of **2** recorded in $CDCl_3$

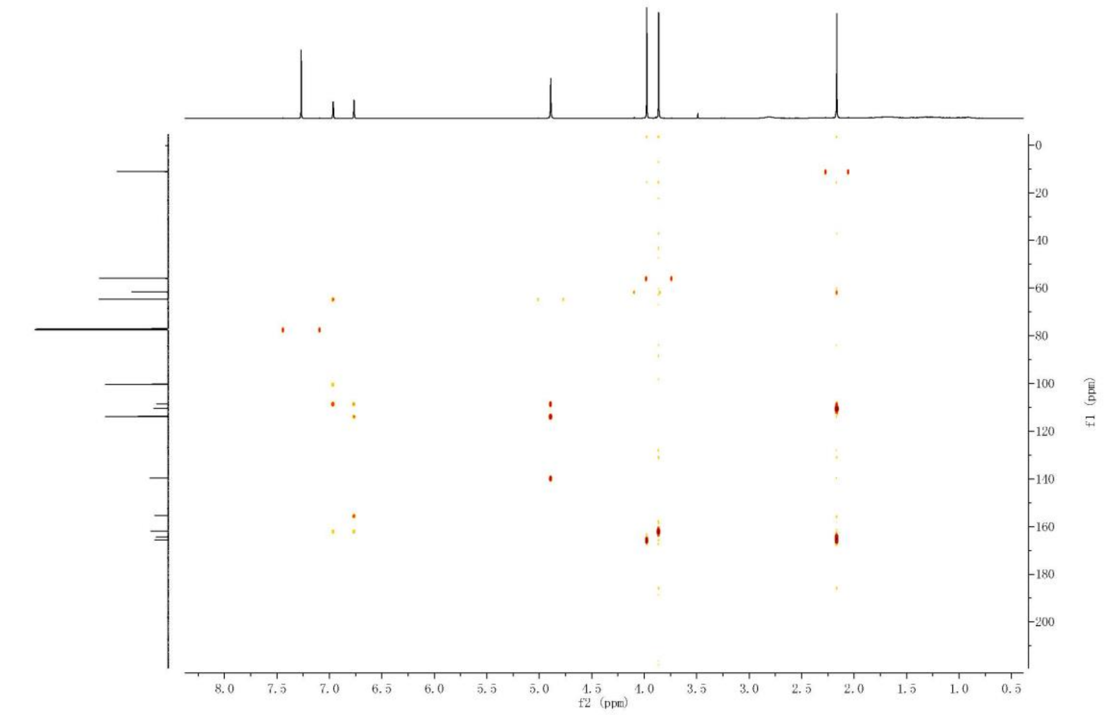

HMBC spectrum of **2** recorded in $CDCl_3$

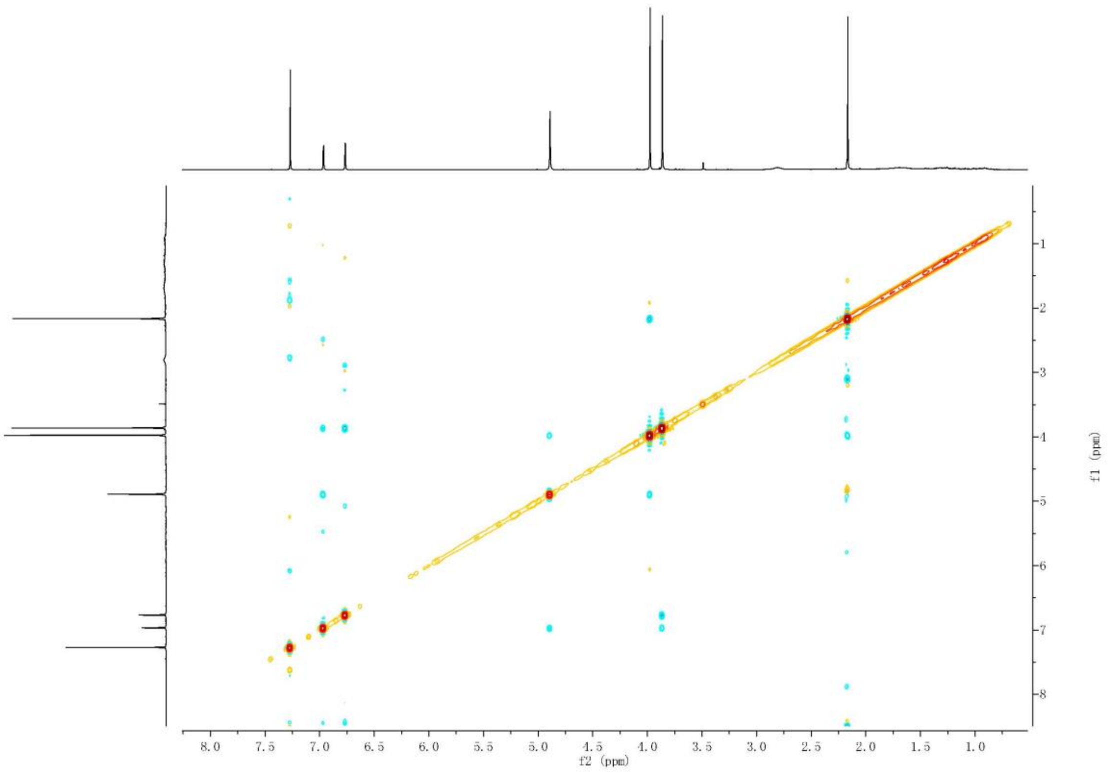

ROESY spectrum of **2** recorded in $CDCl_3$

Data File: E:\百度云同步盘\昆植所\实验室\LC-MS结果\假具苞铃子香\2018-01-31_2018131_Cp-A-4_10.lcd

Elmt	Val.	Min	Max	Elmt	Val.	Min	Max	Elmt	Val.	Min	Max	Use Adduct
H	1	0	300	O	2	0	50	Br	1	0	0	H
C	4	0	150	S	2	0	0					Na
N	3	0	0	Cl	1	0	0					

Error Margin (ppm):	50	DBE Range:	0.0 - 30.0	Electron Ions:	both
HC Ratio:	unlimited	Apply N Rule:	yes	Use MSn Info:	no
Max Isotopes:	all	Isotope RI (%):	1.00	Isotope Res:	10000
MSn Iso RI (%):	75.00	MSn Logic Mode:	AND	Max Results:	800

Event#: 1 MS(E+) Ret. Time : 9.297 Scan# : 1872

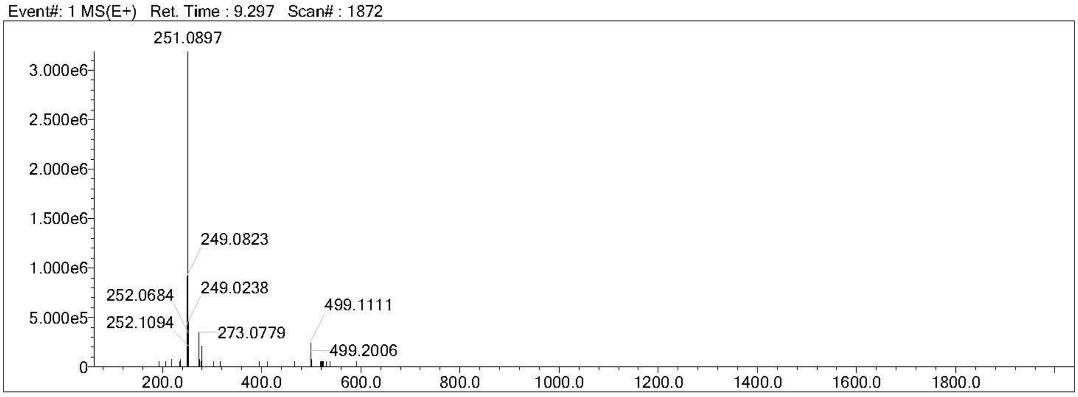

Measured region for 251.0897 m/z

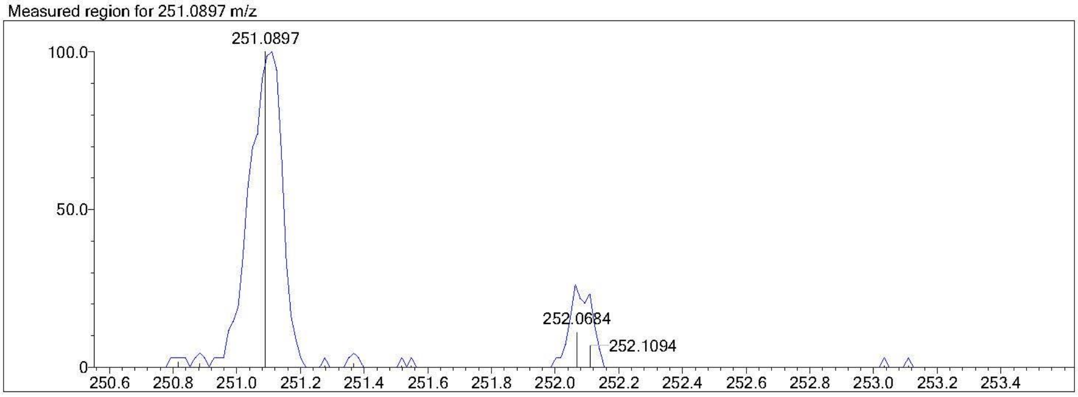

C13 H14 O5 [M+H]+ : Predicted region for 251.0914 m/z

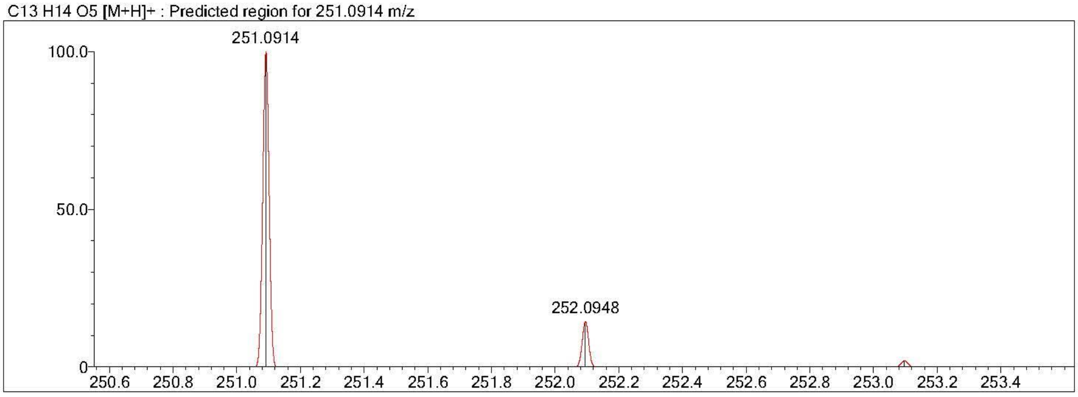

Rank	Score	Formula (M)	Ion	Meas. m/z	Pred. m/z	Df. (mDa)	Df. (ppm)	Iso	DBE
2	28.49	C13 H14 O5	[M+H]+	251.0897	251.0914	-1.7	-6.77	39.40	7.0

HR-ESI-MS spectrum of **2**

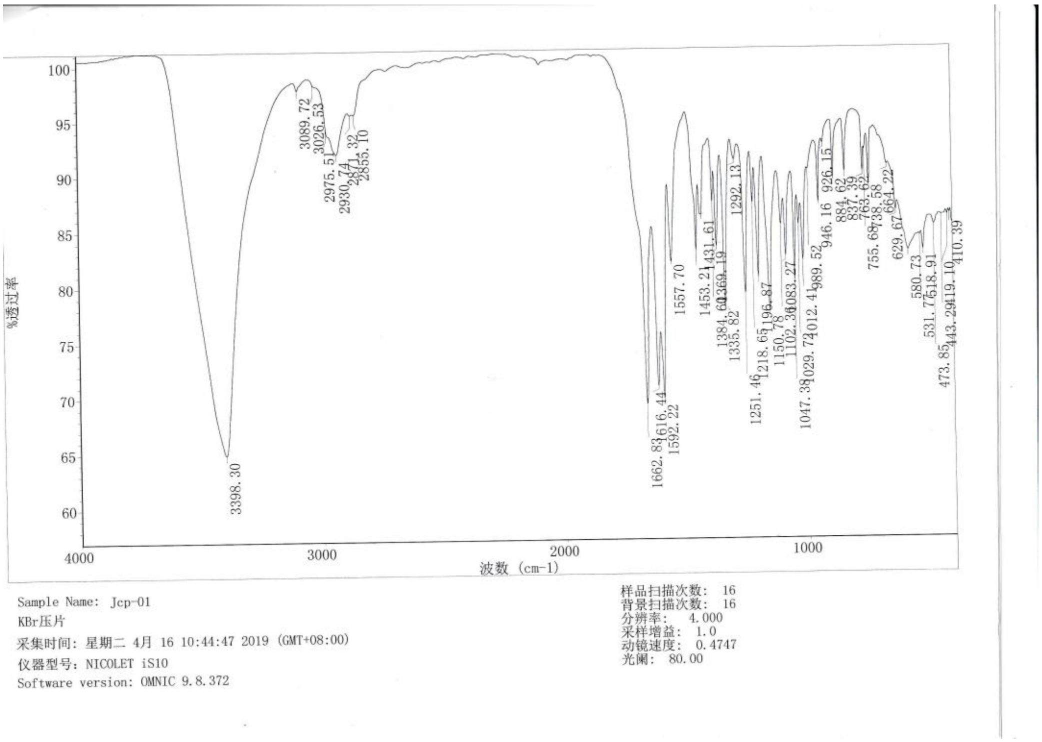

IR spectrum of **2**

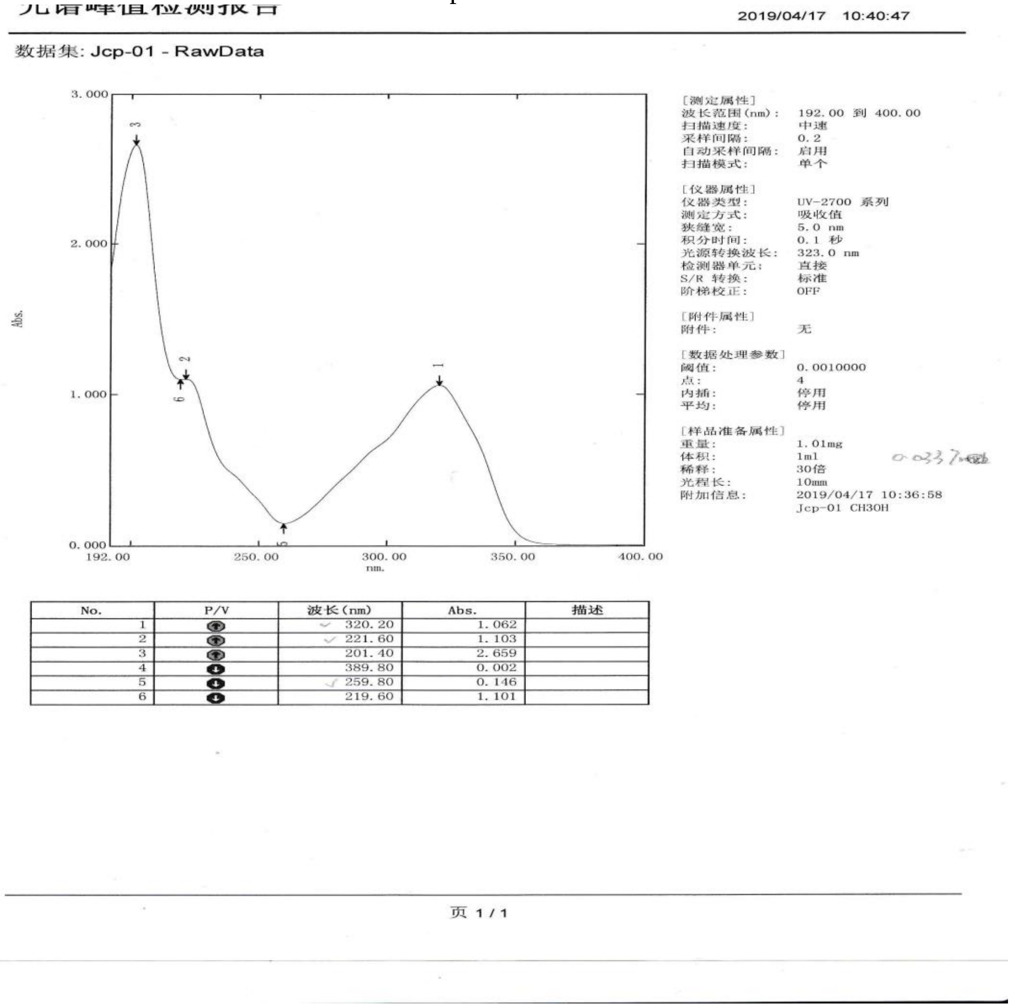

No.	P/V	波长(nm)	Abs.	描述
1	↑	320.20	1.062	
2	↑	221.60	1.103	
3	↑	201.40	2.659	
4	↓	389.80	0.002	
5	↓	259.80	0.146	
6	↓	219.60	1.101	

UV spectrum of **2**

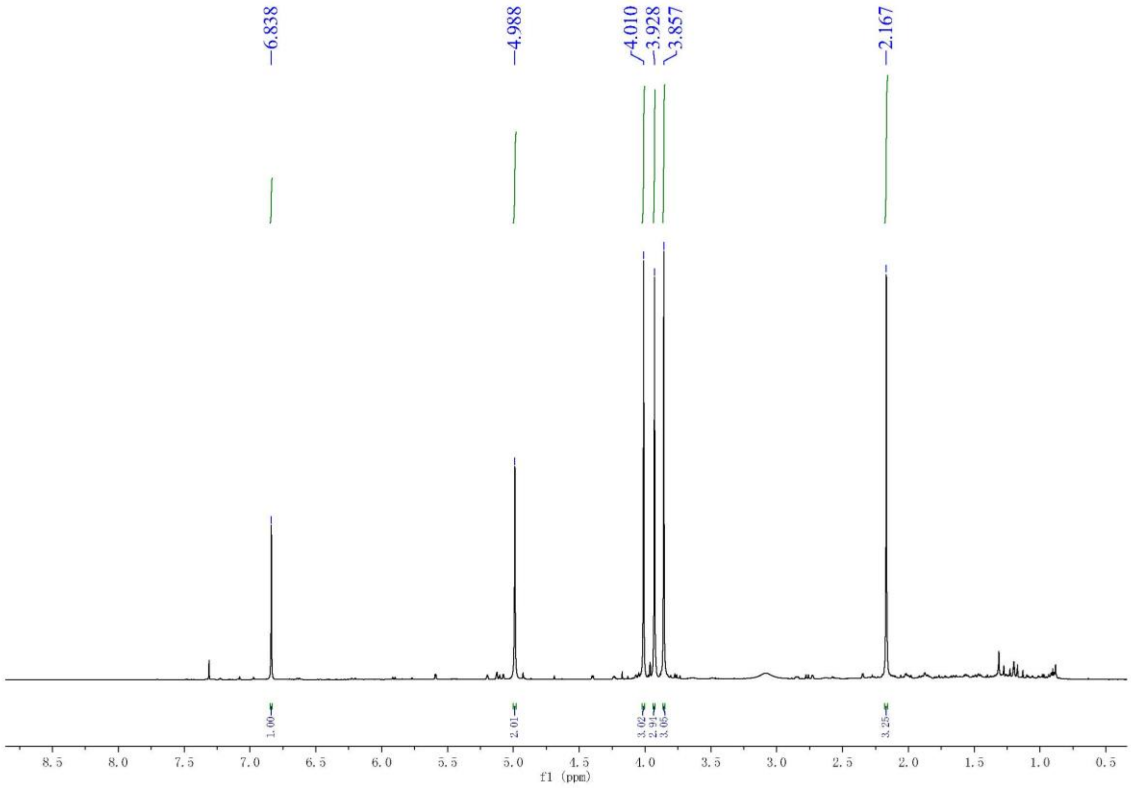

^{1}H-NMR spectrum of **3** recorded in $CDCl_3$ at 600MHz

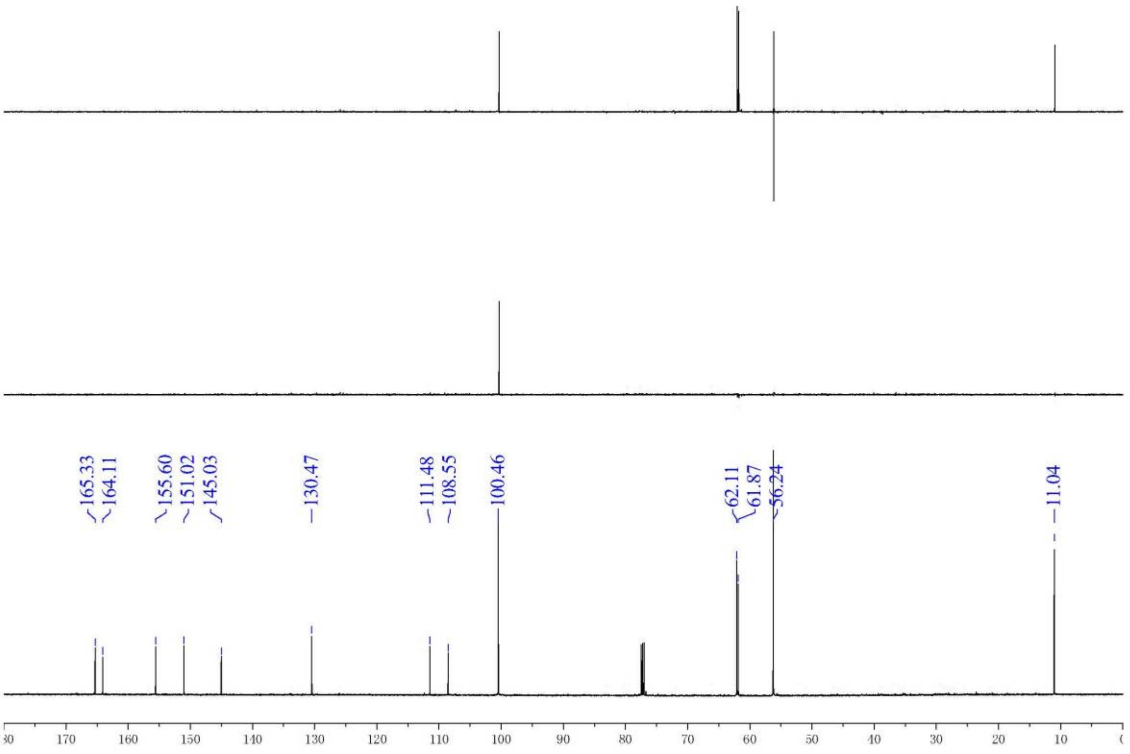

^{13}C-NMR spectrum of **3** recorded in $CDCl_3$ at 150MHz

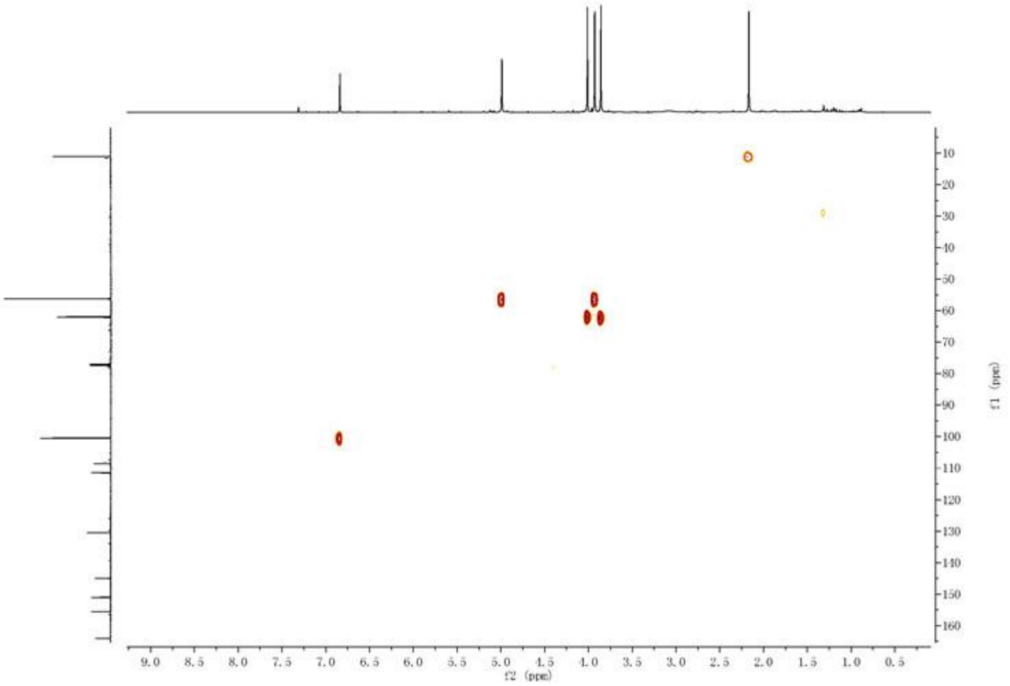

HSQC spectrum of **3** recorded in $CDCl_3$

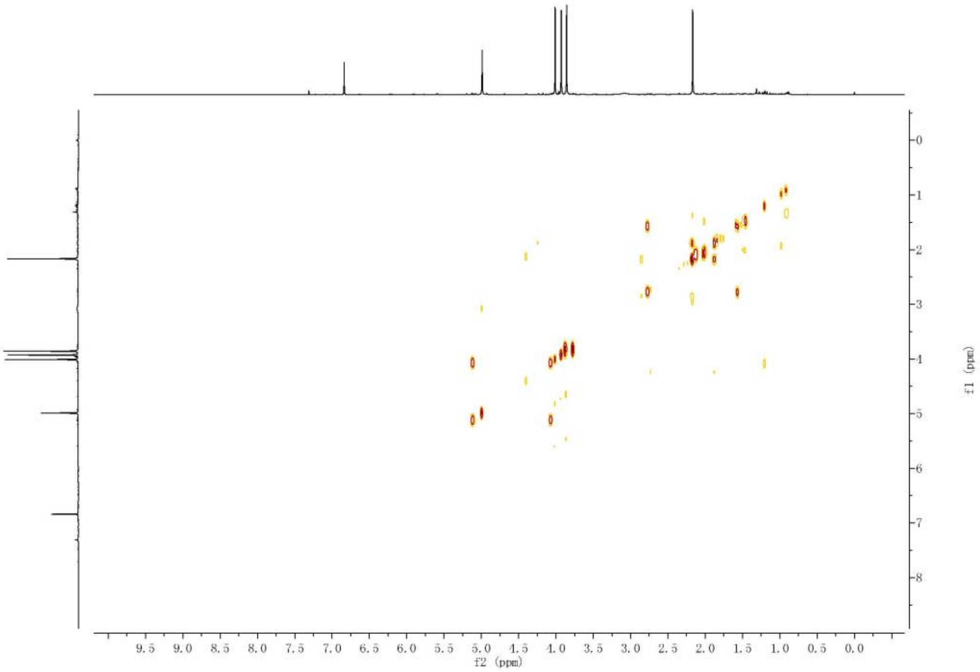

^1H-^1H COSY spectrum of **3** recorded in $CDCl_3$

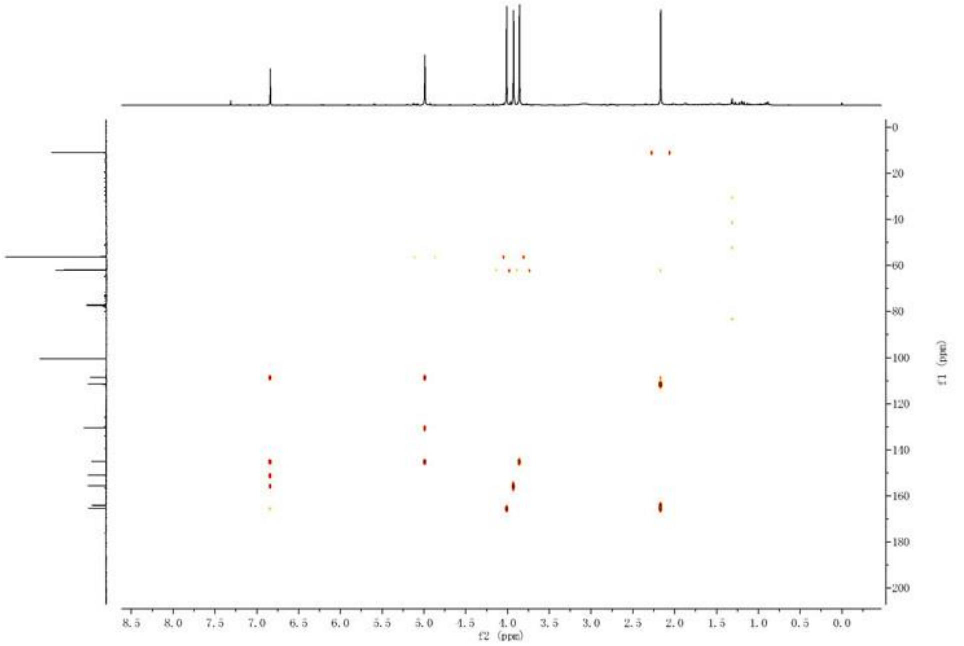

HMBC spectrum of **3** recorded in $CDCl_3$

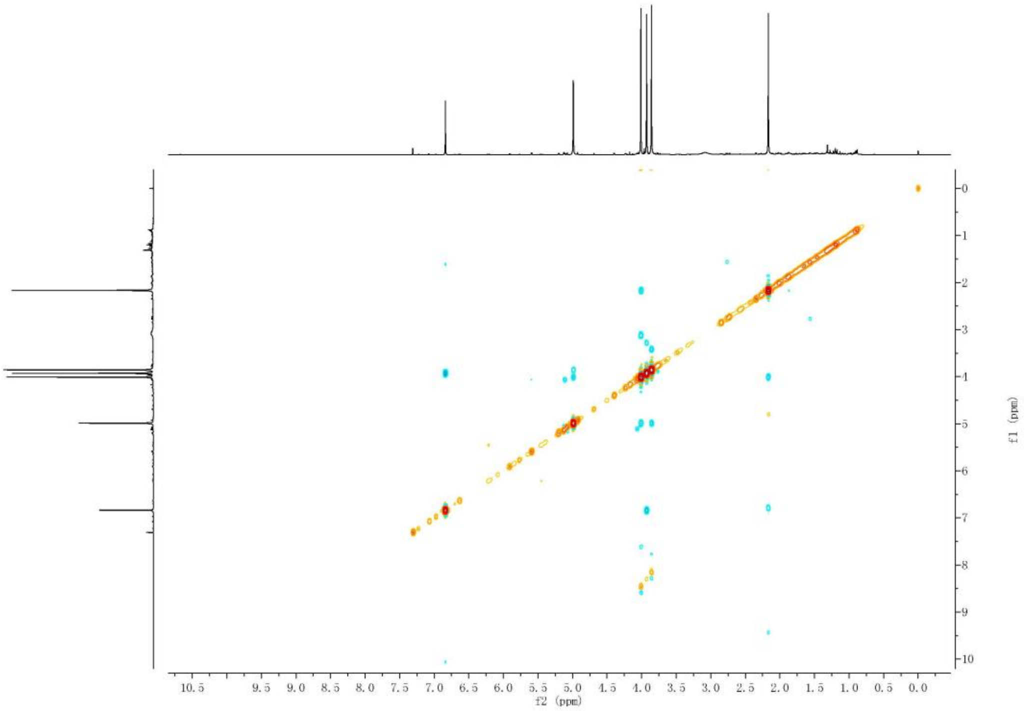

ROESY spectrum of **3** recorded in $CDCl_3$

Formula Predictor Report - Data006.lcd

Data File: E:\百度云同步盘\昆植所\实验室\LC-MS结果\假具苞铃子香\Cp-A-5,6,7合\CpA-5-1,2合\Data006.lcd

Elmt	Val.	Min	Max	Elmt	Val.	Min	Max	Elmt	Val.	Min	Max	Use Adduct
H	1	0	300	O	2	0	50	Br	1	0	0	H
C	4	0	150	S	2	0	0					Na
N	3	0	0	Cl	1	0	0					

Error Margin (ppm):	50	DBE Range:	0.0 - 30.0	Electron Ions:	both
HC Ratio:	unlimited	Apply N Rule:	yes	Use MSn Info:	no
Max Isotopes:	all	Isotope RI (%):	1.00	Isotope Res:	10000
MSn Iso RI (%):	75.00	MSn Logic Mode:	AND	Max Results:	800

Event#: 1 MS(E+) Ret. Time : 6.523 Scan# : 1305

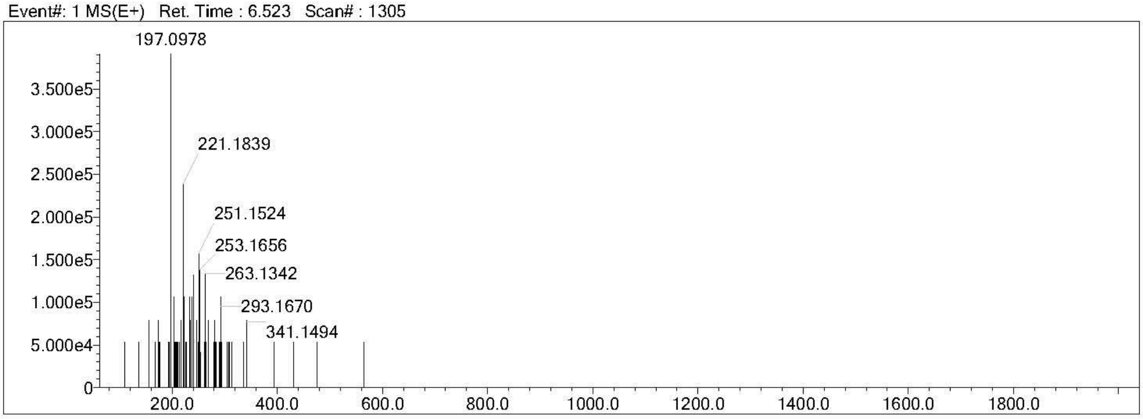

Measured region for 281.1039 m/z

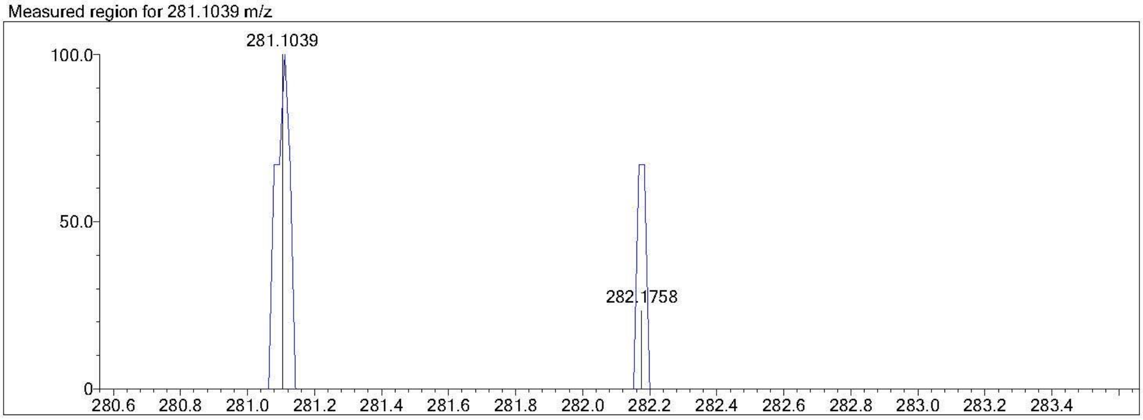

C14 H16 O6 [M+H]+ : Predicted region for 281.1020 m/z

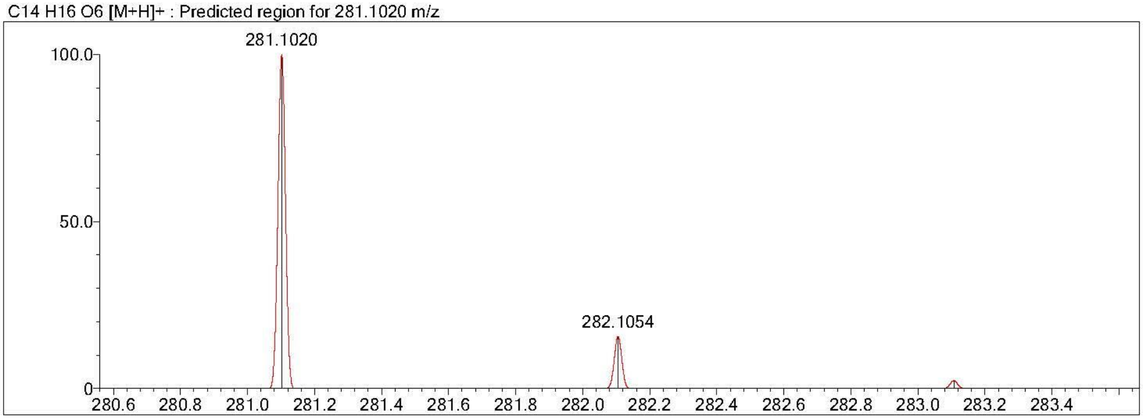

Rank	Score	Formula (M)	Ion	Meas. m/z	Pred. m/z	Df. (mDa)	Df. (ppm)	Iso	DBE
1	0.00	C14 H16 O6	[M+H]+	281.1039	281.1020	1.9	6.76	0.00	7.0

HR-ESI-MS spectrum of **3**

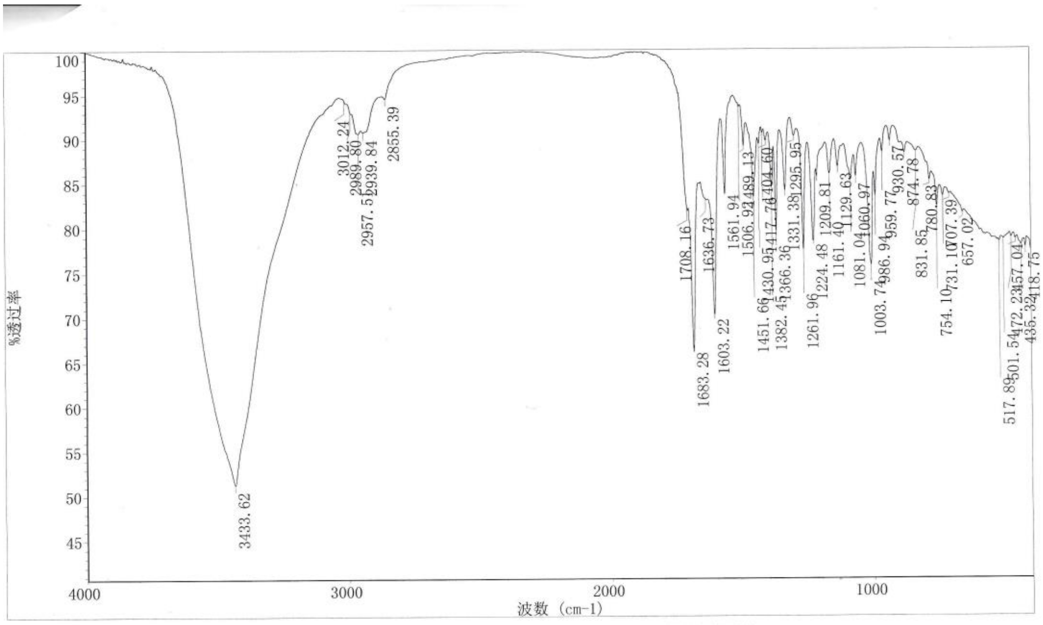

Sample Name: Jcp-02
KBr压片
采集时间: 星期二 4月 16 11:11:35 2019 (GMT+08:00)
仪器型号: NICOLET iS10
Software version: OMNIC 9.8.372

样品扫描次数: 64
背景扫描次数: 16
分辨率: 4.000
采样增益: 1.0
动镜速度: 0.4747
光阑: 80.00

IR spectrum of **3**

光谱峰值检测报告 2019/04/17 10:46:00

数据集: Jcp-02 - RawData

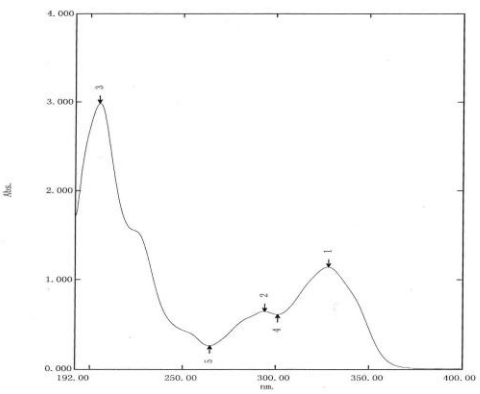

[测定属性]
波长范围(nm): 192.00 到 400.00
扫描速度: 中速
采样间隔: 0.2
自动采样间隔: 启用
扫描模式: 单个

[仪器属性]
仪器类型: UV-2700 系列
测定方式: 吸收值
狭缝宽: 5.0 nm
积分时间: 0.1 秒
光源转换波长: 323.0 nm
检测器单元: 直接
S/R 转换: 标准
阶梯校正: OFF

[附件属性]
附件: 无

[数据处理参数]
阈值: 0.0010000
点: 4
内插: 停用
平均: 停用

[样品准备属性]
重量: 0.97mg
体积: 1ml
稀释: 30倍
光程长: 10mm
附加信息: 2019/04/17 10:42:18
Jcp-02 CH3OH

No.	P/V	波长(nm)	Abs.	描述
1	↑	328.00	1.142	
2	↑	293.60	0.646	
3	↑	205.00	2.982	
4	↓	300.80	0.611	
5	↓	264.20	0.260	

页 1 / 1

UV spectrum of **3**

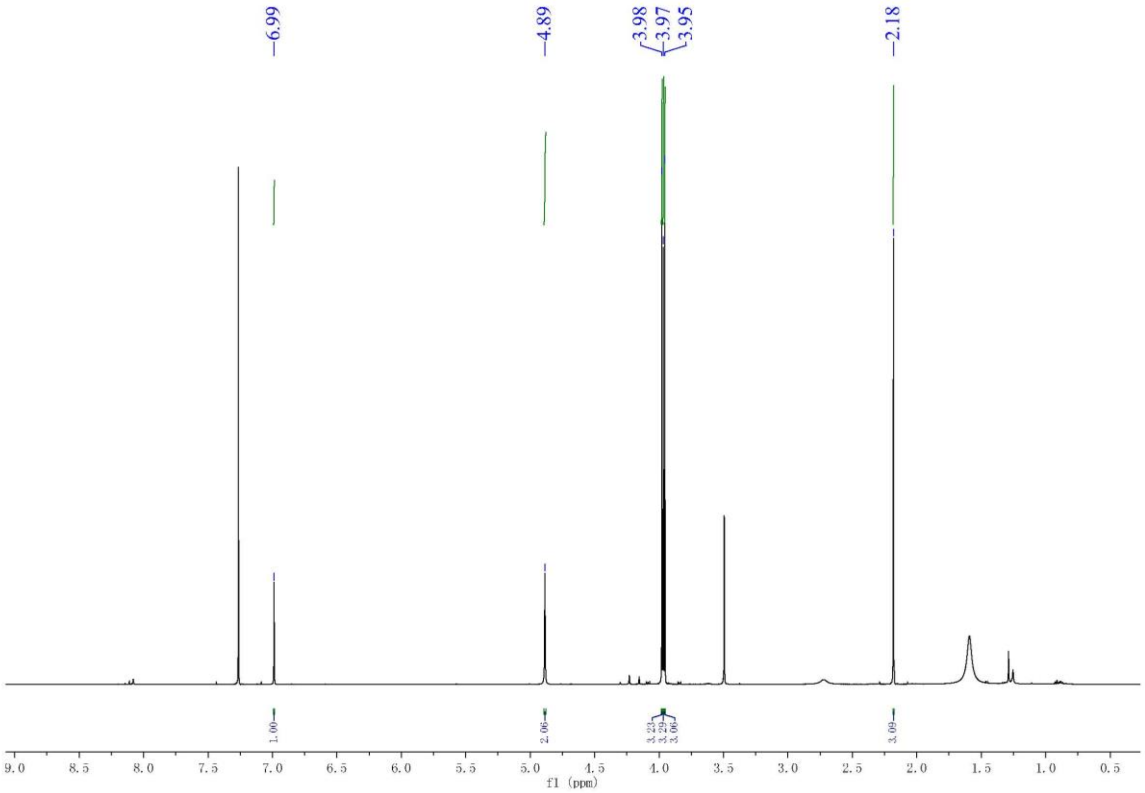

^{1}H-NMR spectrum of **4** recorded in $CDCl_3$ at 600MHz

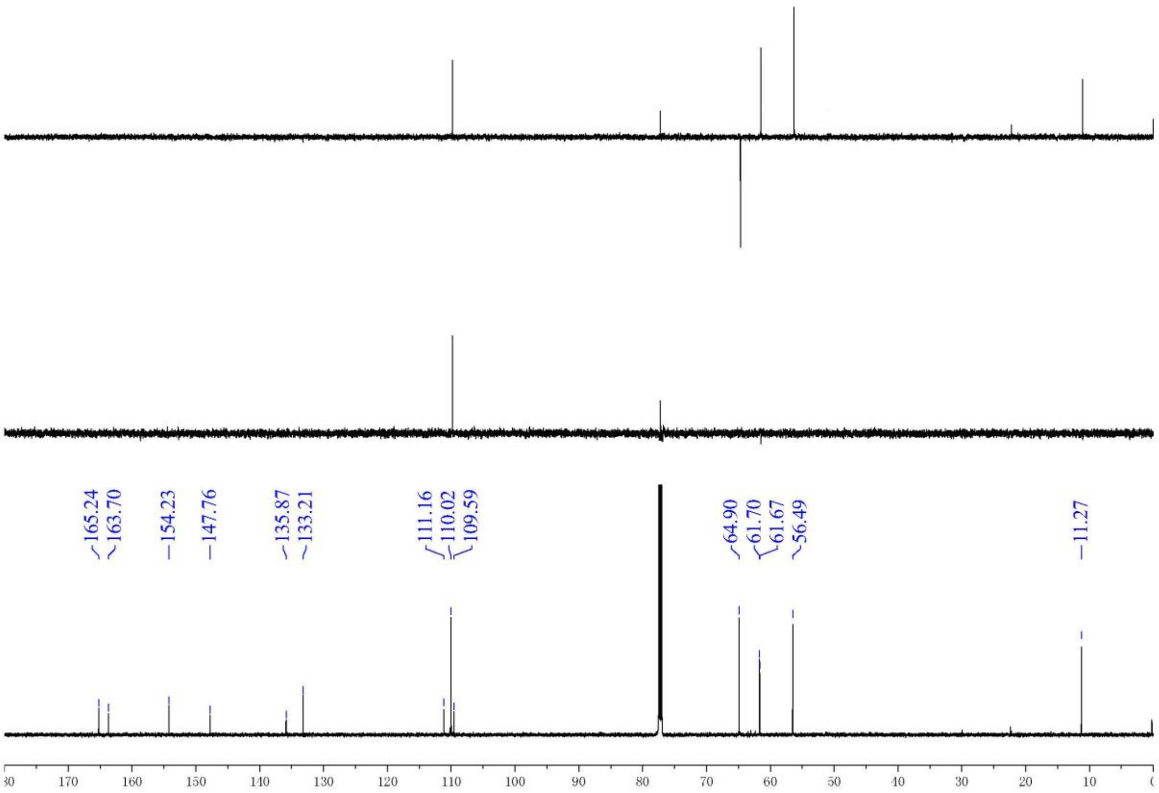

^{13}C-NMR spectrum of **4** recorded in $CDCl_3$ at 150MHz

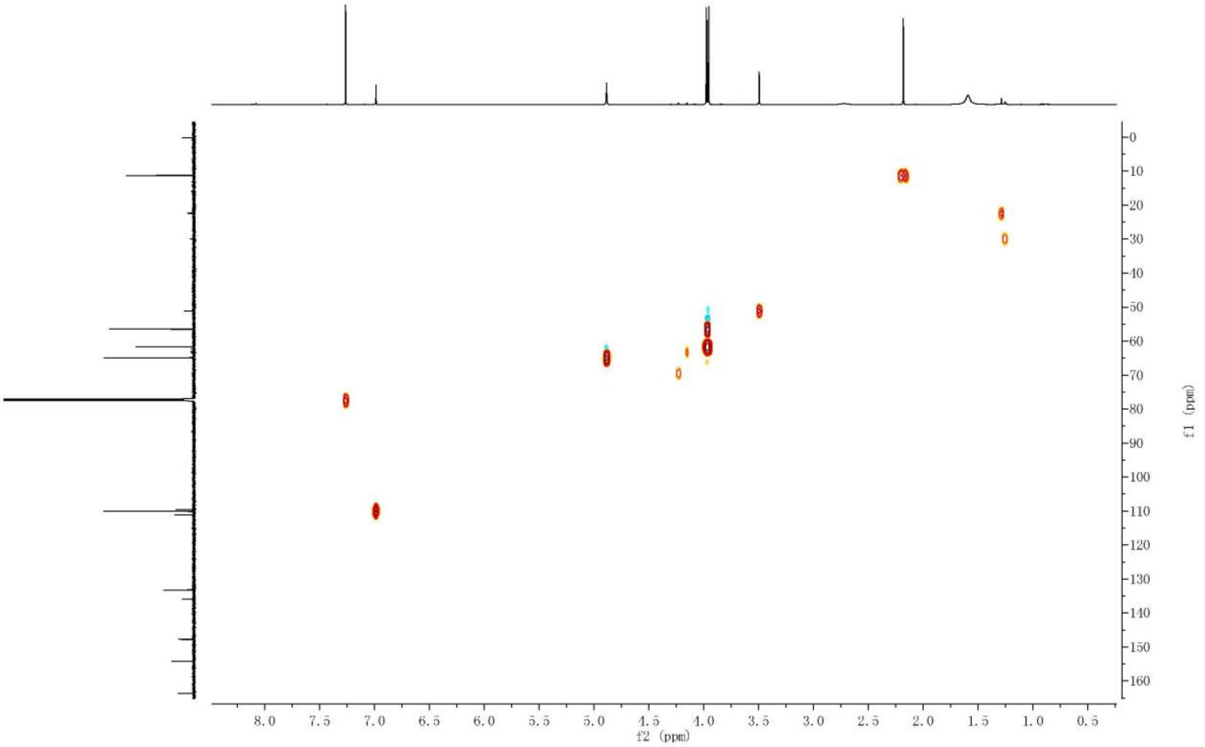

HSQC spectrum of **4** recorded in $CDCl_3$

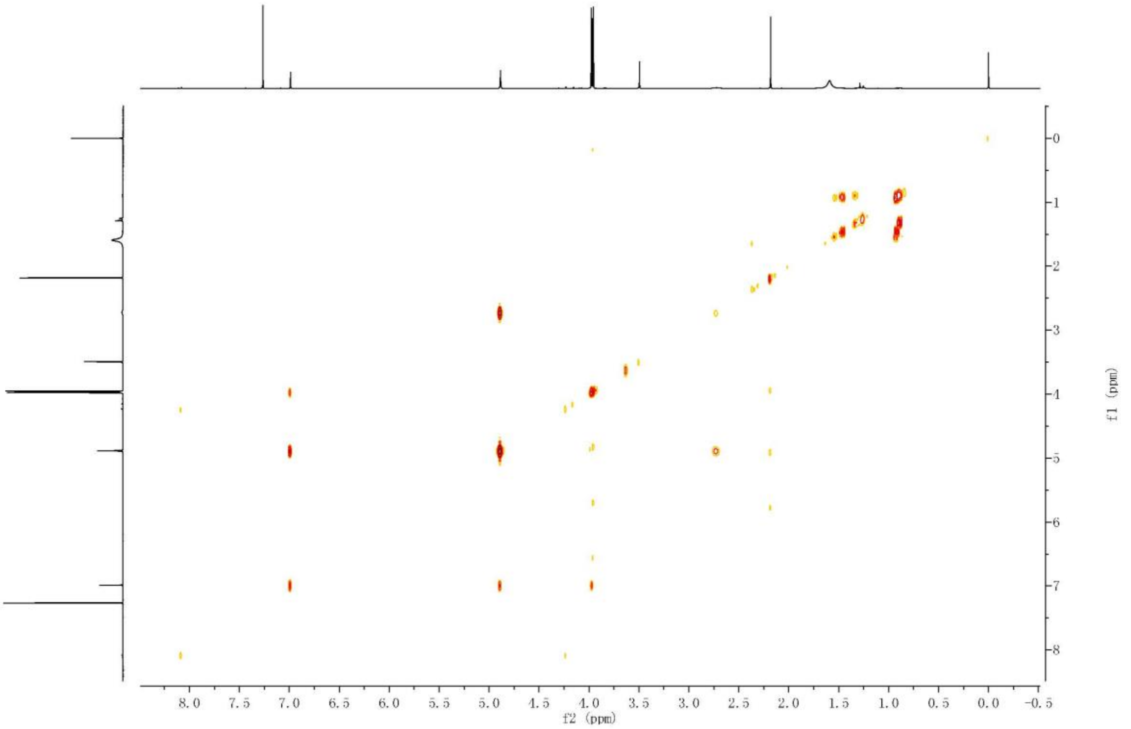

^{1}H-^{1}H COSY spectrum of **4** recorded in $CDCl_3$

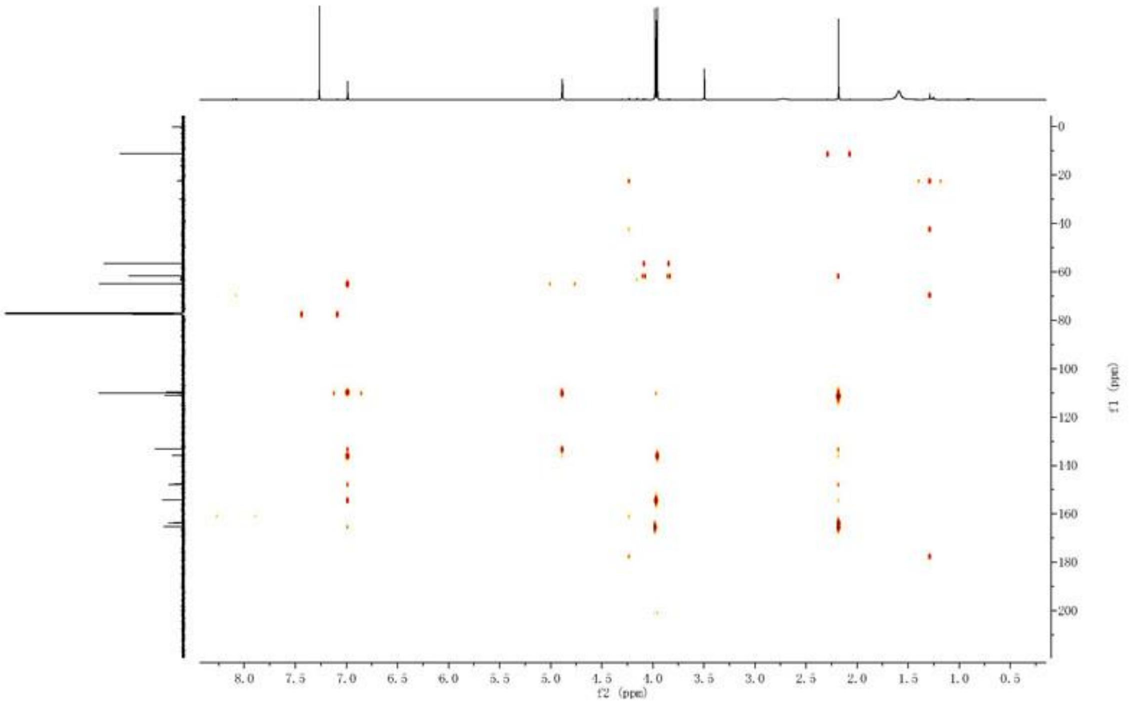

HMBC spectrum of **4** recorded in $CDCl_3$

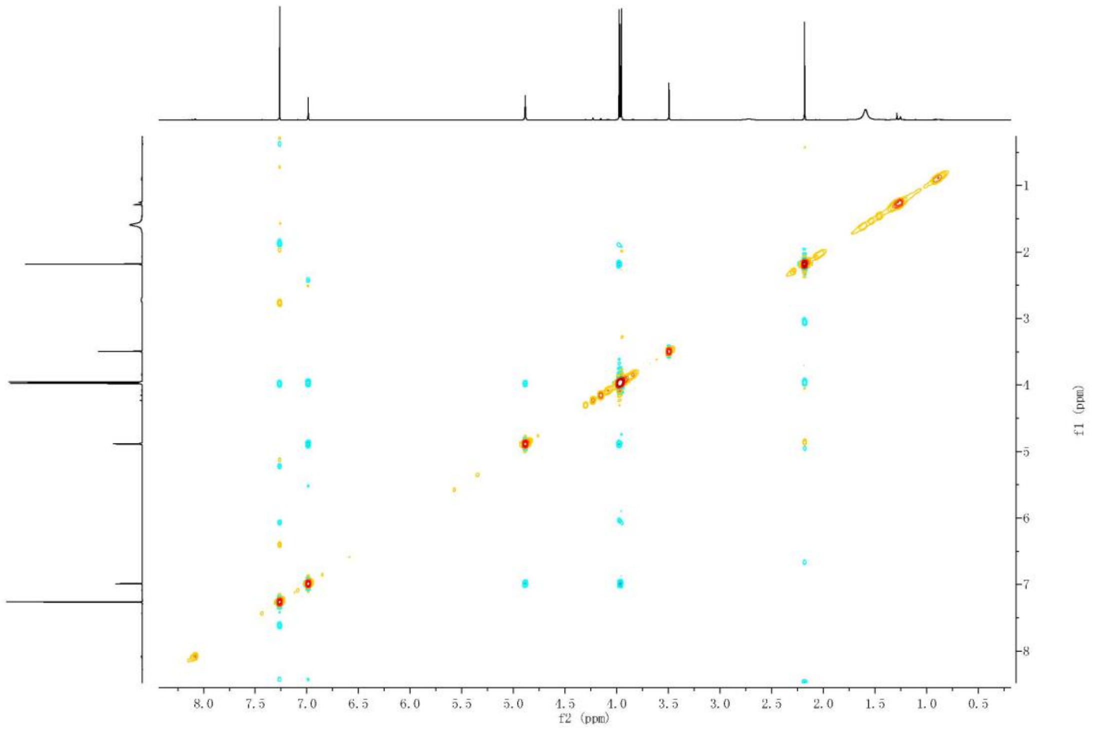

ROESY spectrum of **4** recorded in $CDCl_3$

Data File: E:\百度云同步盘\昆植所\实验室\LC-MS结果\齿唇铃子香\CoA-6各部分\Co-A-6-2各部分\CoA-623各部分\CoA-6232_2018-06-05LCMS_2...

Elmt	Val.	Min	Max	Elmt	Val.	Min	Max	Elmt	Val.	Min	Max	Use Adduct
H	1	0	300	O	2	0	50	Br	1	0	0	H
C	4	0	150	S	2	0	0					Na
N	3	0	0	Cl	1	0	0					

Error Margin (ppm):	100	DBE Range:	0.0 - 30.0	Electron Ions:	both
HC Ratio:	unlimited	Apply N Rule:	yes	Use MSn Info:	no
Max Isotopes:	all	Isotope RI (%):	1.00	Isotope Res:	10000
MSn Iso RI (%):	75.00	MSn Logic Mode:	AND	Max Results:	800

Event#: 1 MS(E+) Ret. Time : 7.782 Scan# : 1588

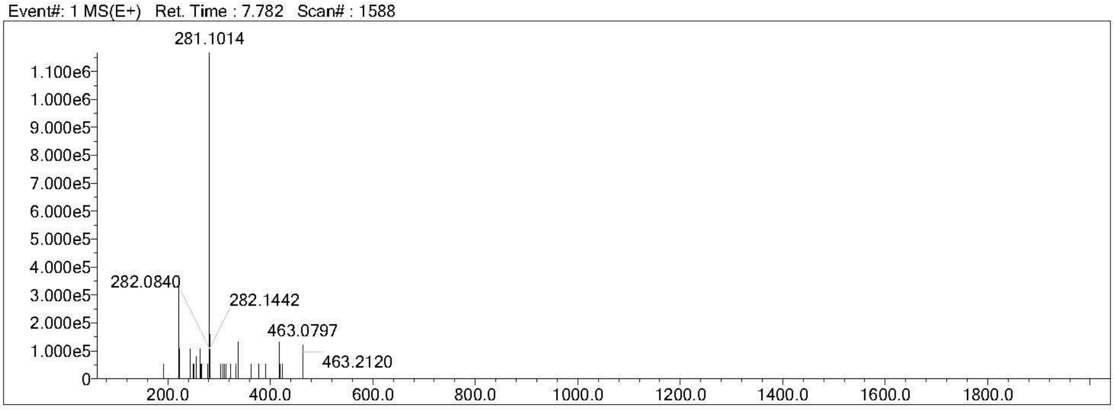

Measured region for 281.1014 m/z

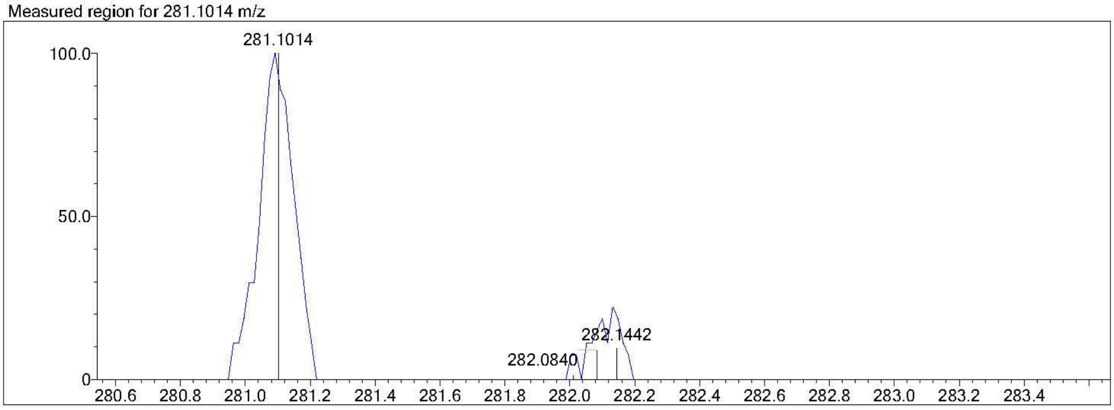

C14 H16 O6 [M+H]+ : Predicted region for 281.1020 m/z

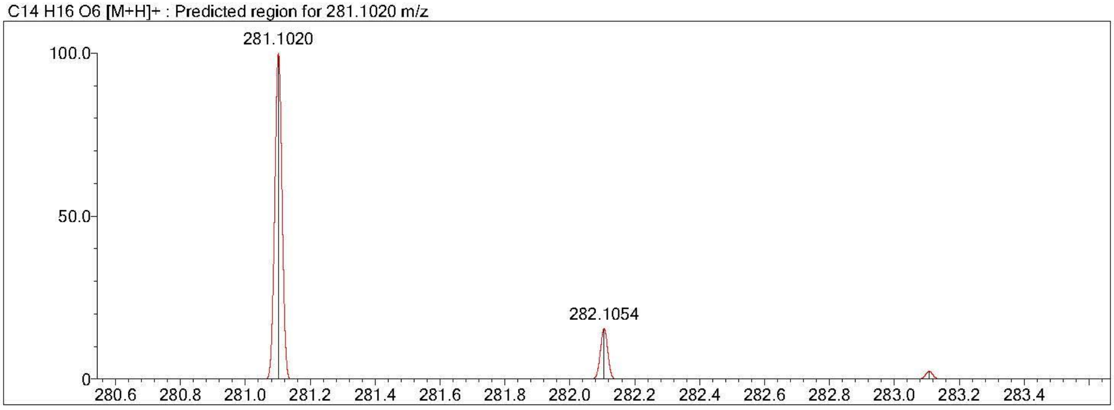

Rank	Score	Formula (M)	Ion	Meas. m/z	Pred. m/z	Df. (mDa)	Df. (ppm)	Iso	DBE
1	36.33	C14 H16 O6	[M+H]+	281.1014	281.1020	-0.6	-2.13	37.39	7.0

HR-ESI-MS spectrum of **4**

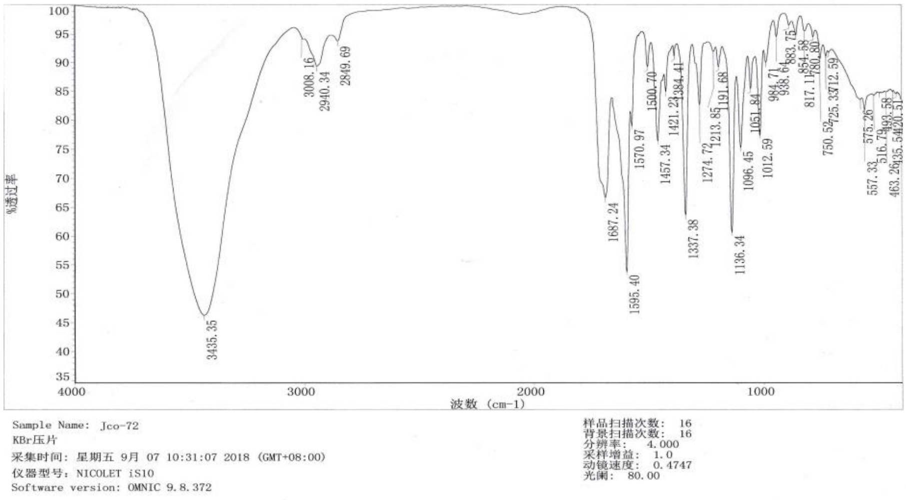

IR spectrum of **4**

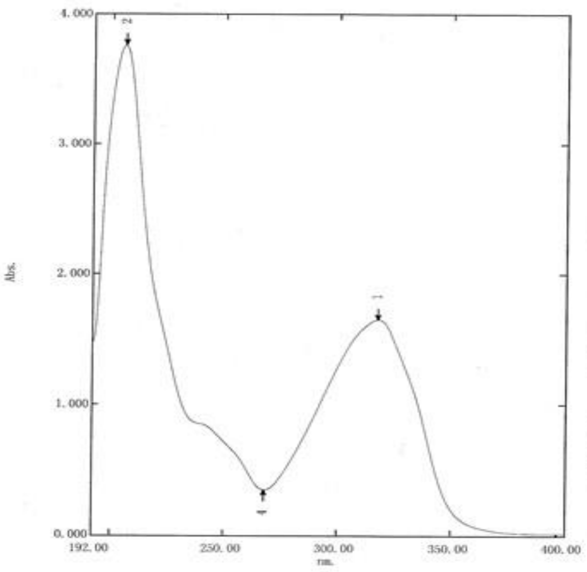

光谱峰值检测报告

2019/04/17 10:52:00

数据集: Jco-72 - RawData

[测定属性]
波长范围(nm): 192.00 到 400.00
扫描速度: 中速
采样间隔: 0.2
自动采样间隔: 启用
扫描模式: 单个

[仪器属性]
仪器类型: UV-2700 系列
测定方式: 吸收值
狭缝宽: 5.0 nm
积分时间: 0.1 秒
光源转换波长: 323.0 nm
检测器单元: 直接
S/R 转换: 标准
阶梯校正: OFF

[附件属性]
附件: 无

[数据处理参数]
阈值: 0.0010000
点: 4
内插: 停用
平均: 停用

[样品准备属性]
重量: 1.19mg
体积: 1ml
稀释: 30倍
光程长: 10mm
附加信息: 2019/04/17 10:47:36
Jco-72 CH3OH

No.	P/V	波长(nm)	Abs.	描述
1	P	317.60	1.654	
2	P	205.80	3.759	
3	V	396.80	0.011	
4	V	267.80	0.346	

页 1 / 1

UV spectrum of **4**

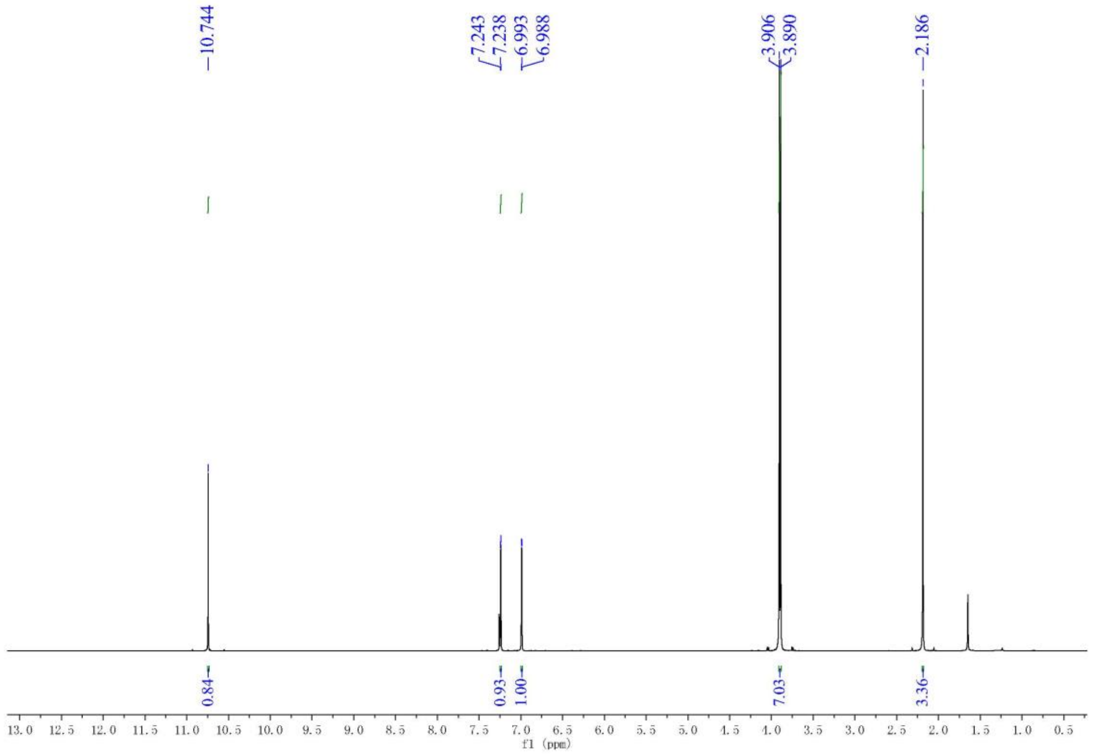

^{1}H-NMR spectrum of **5** recorded in $CDCl_3$ at 500MHz

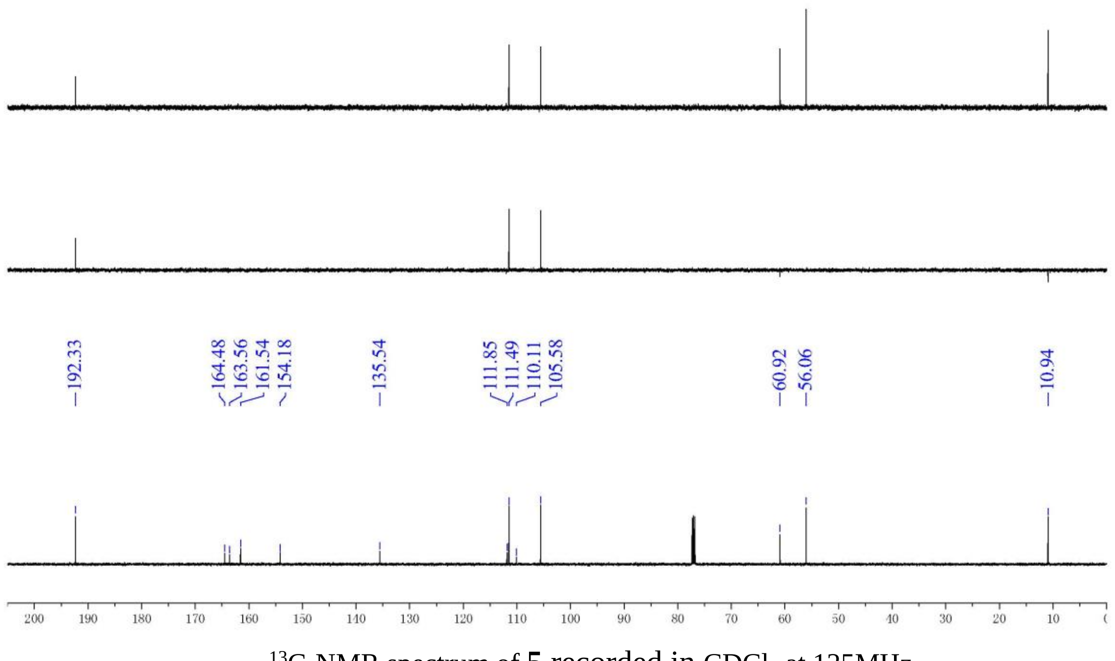

^{13}C-NMR spectrum of **5** recorded in $CDCl_3$ at 125MHz

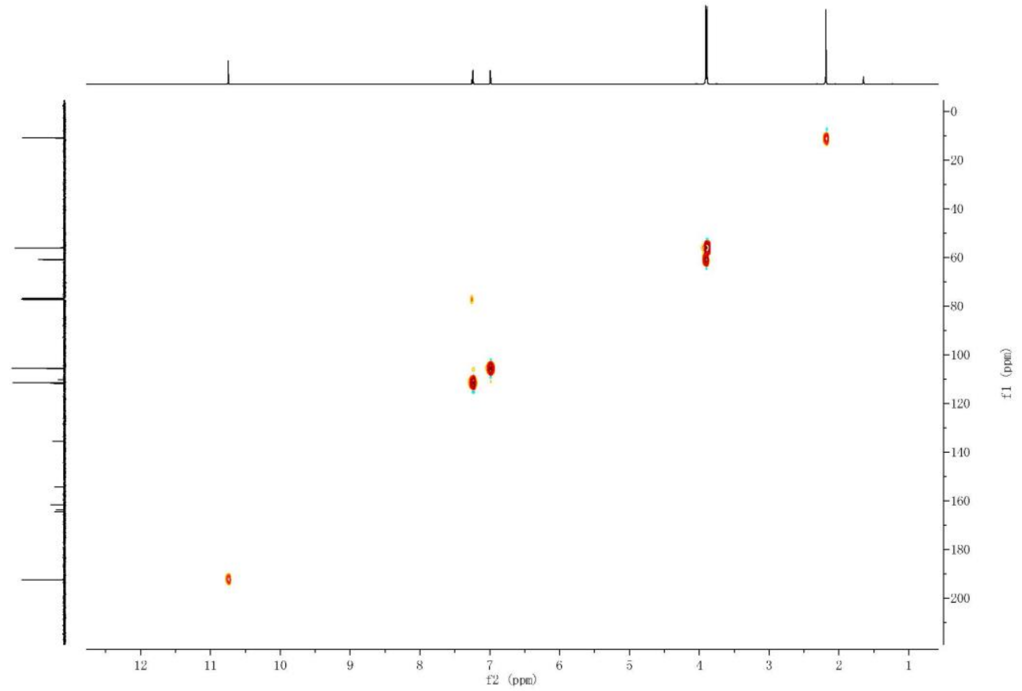

HSQC spectrum of **5** recorded in CDCl3

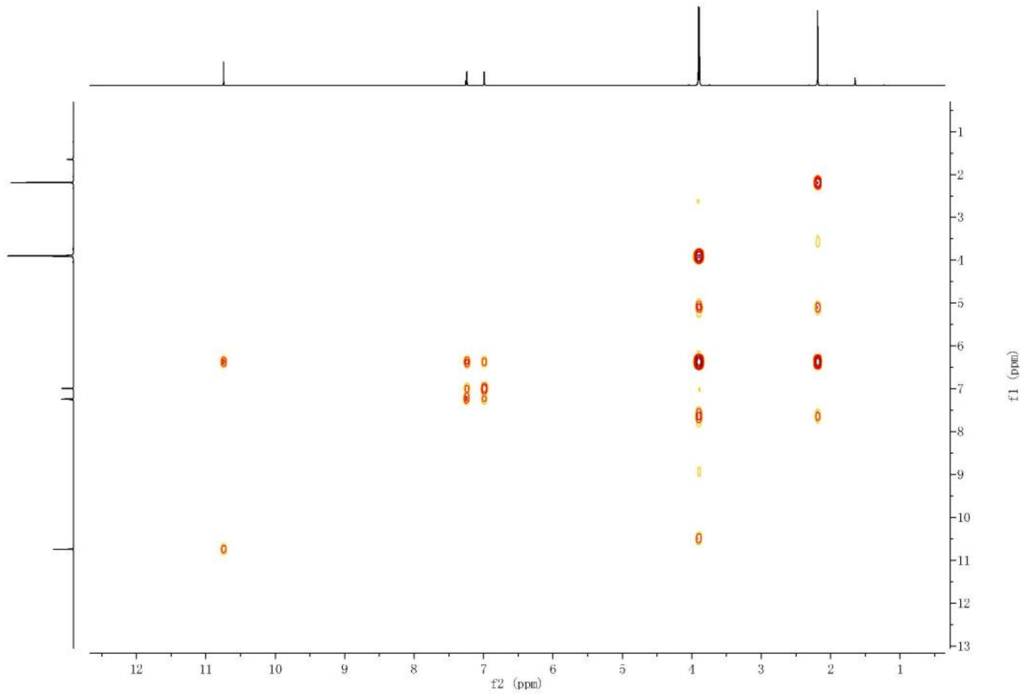

^{1}H-^{1}H COSY spectrum of **5** recorded in $CDCl_3$

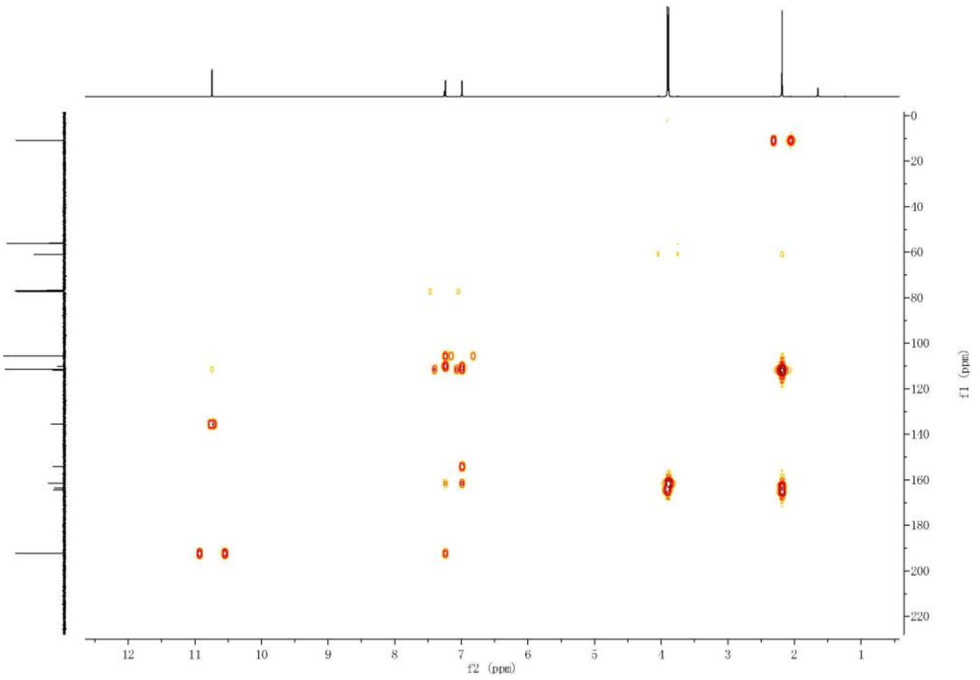

HMBC spectrum of **5** recorded in $CDCl_3$

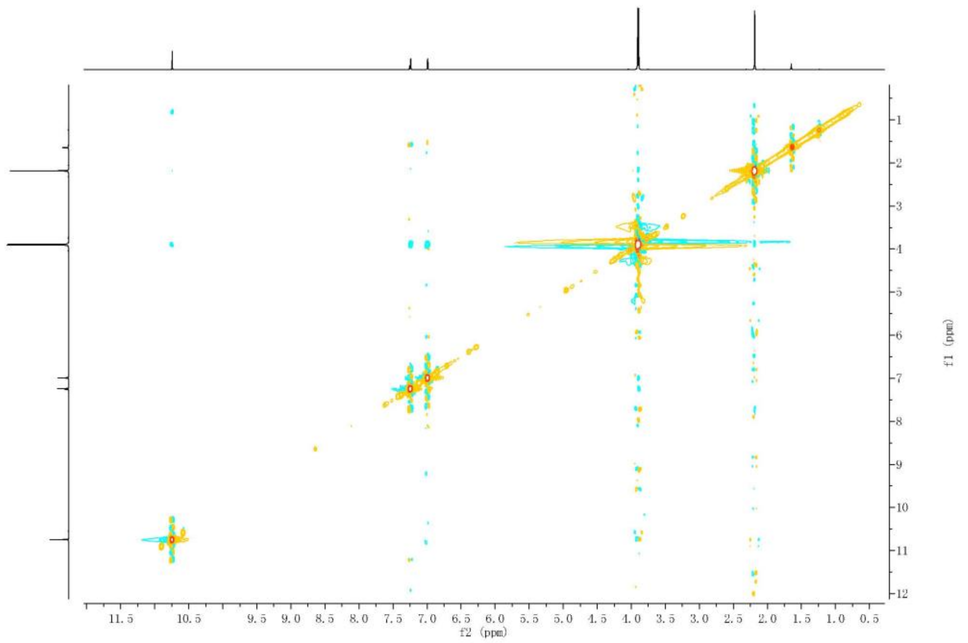

ROESY spectrum of **5** recorded in $CDCl_3$

Formula Predictor Report - 2017-07-21 LCMS_2017721_Cpx-A-6316_7.lcd Page 1 of 1

Data File: E:\百度云同步盘\昆植所\实验室\LC-MS结果\先花铃子香\Cpx-A-6~7合并各部分\Cpx-A-6-3各部分\Cpx-A-6-3-1各部分\2017-07-21 LCMS...

Elmt	Val.	Min	Max	Elmt	Val.	Min	Max	Elmt	Val.	Min	Max	Use Adduct
H	1	0	300	O	2	0	50	Br	1	0	0	H
C	4	0	150	S	2	0	0					Na
N	3	0	10	Cl	1	0	0					

Error Margin (ppm):	50	DBE Range:	0.0 - 30.0	Electron Ions:	both
HC Ratio:	unlimited	Apply N Rule:	yes	Use MSn Info:	no
Max Isotopes:	all	Isotope RI (%):	1.00	Isotope Res:	10000
MSn Iso RI (%):	75.00	MSn Logic Mode:	AND	Max Results:	800

Event#: 1 MS(E+) Ret. Time : 8.382 Scan# : 1734

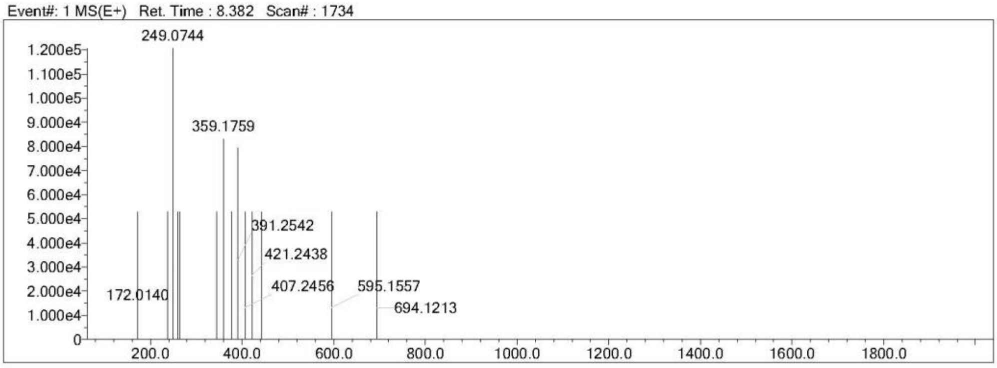

Measured region for 249.0744 m/z

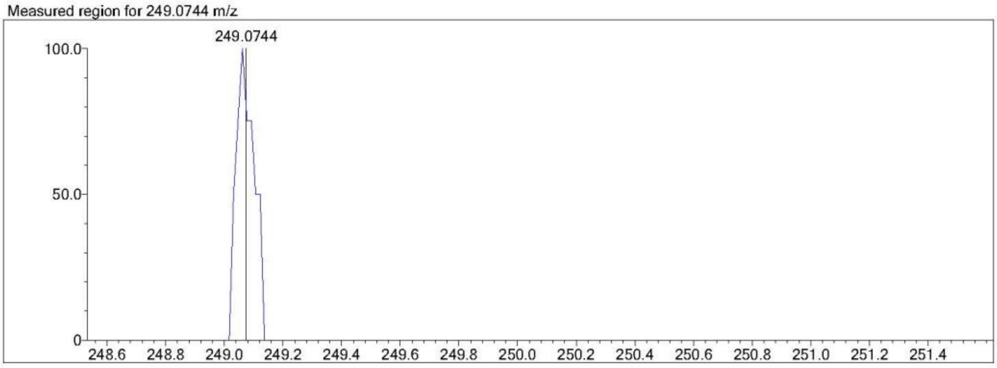

C13 H12 O5 [M+H]+ : Predicted region for 249.0758 m/z

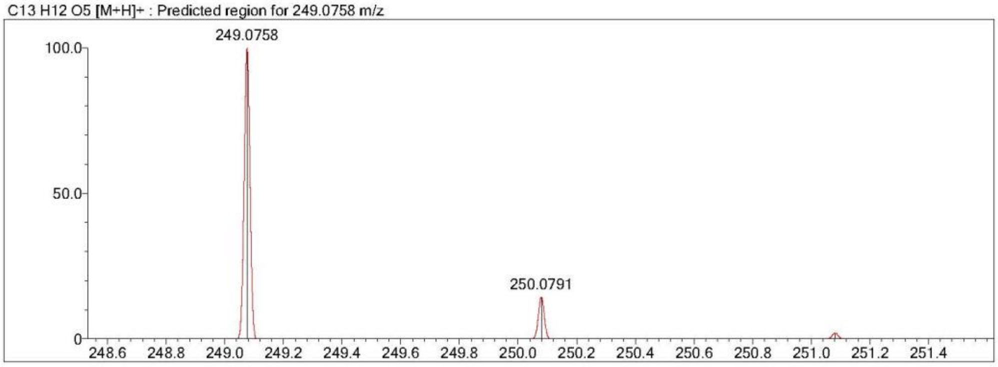

Rank	Score	Formula (M)	Ion	Meas. m/z	Pred. m/z	Df. (mDa)	Df. (ppm)	Iso	DBE
7	0.00	C13 H12 O5	[M+H]+	249.0744	249.0758	-1.4	-5.62	0.00	8.0

HR-ESI-MS spectrum of **5**

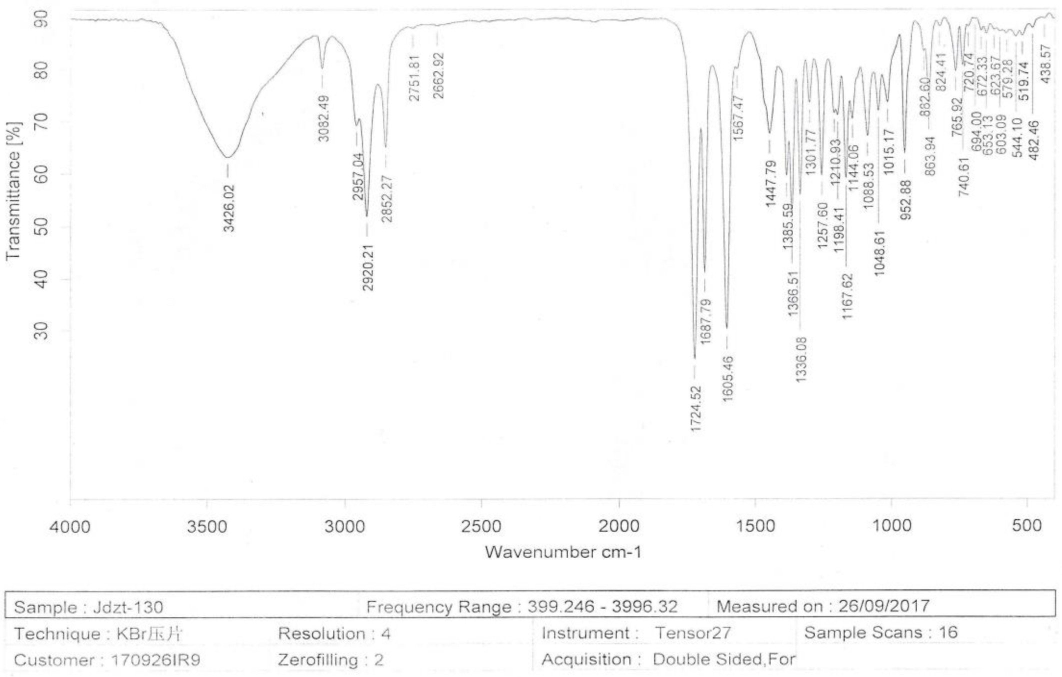

IR spectrum of **5**

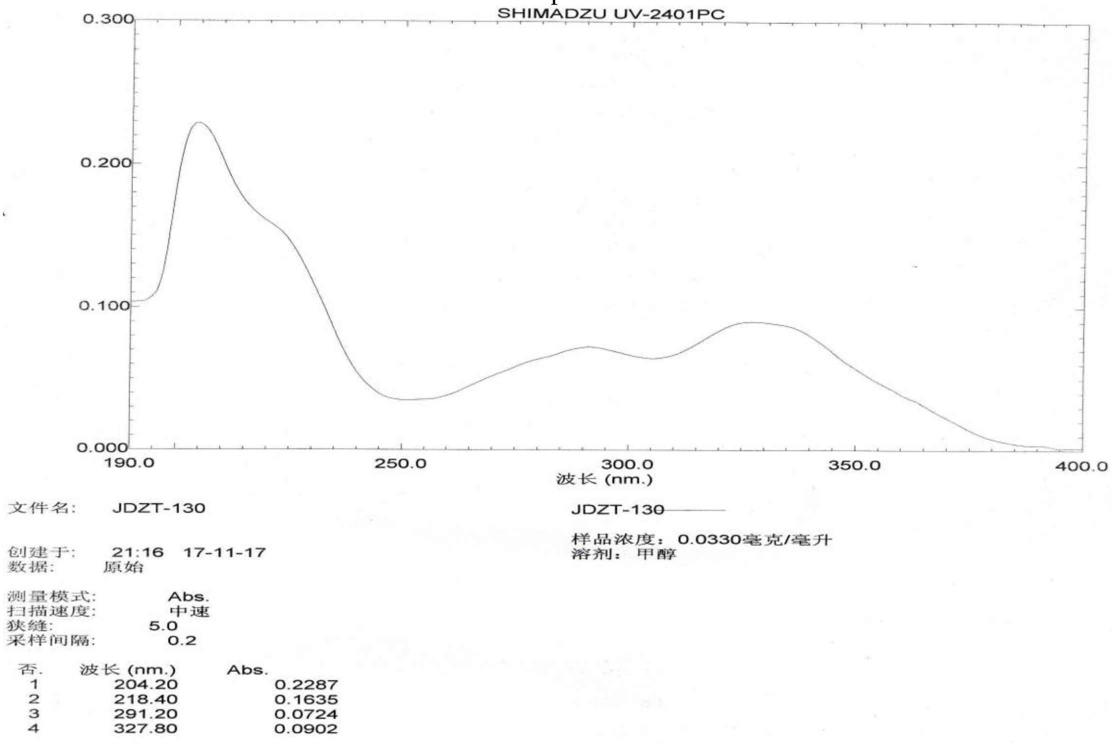

UV spectrum of **5**

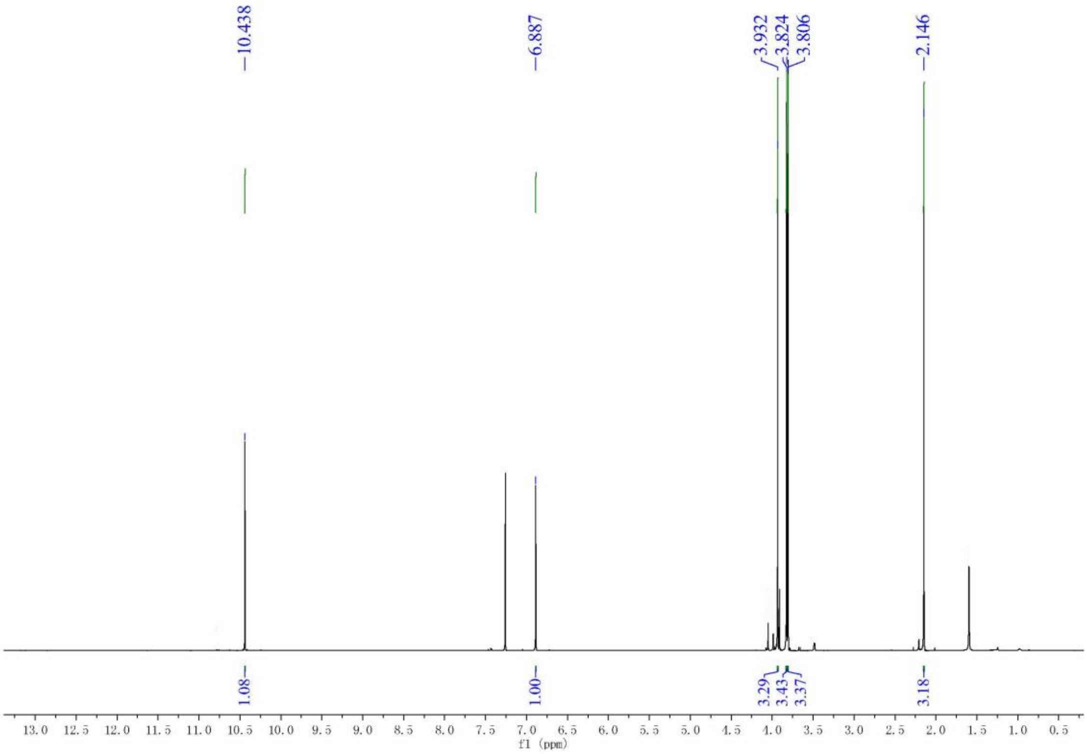

^{1}H-NMR spectrum of **6** recorded in $CDCl_3$ at 500MHz

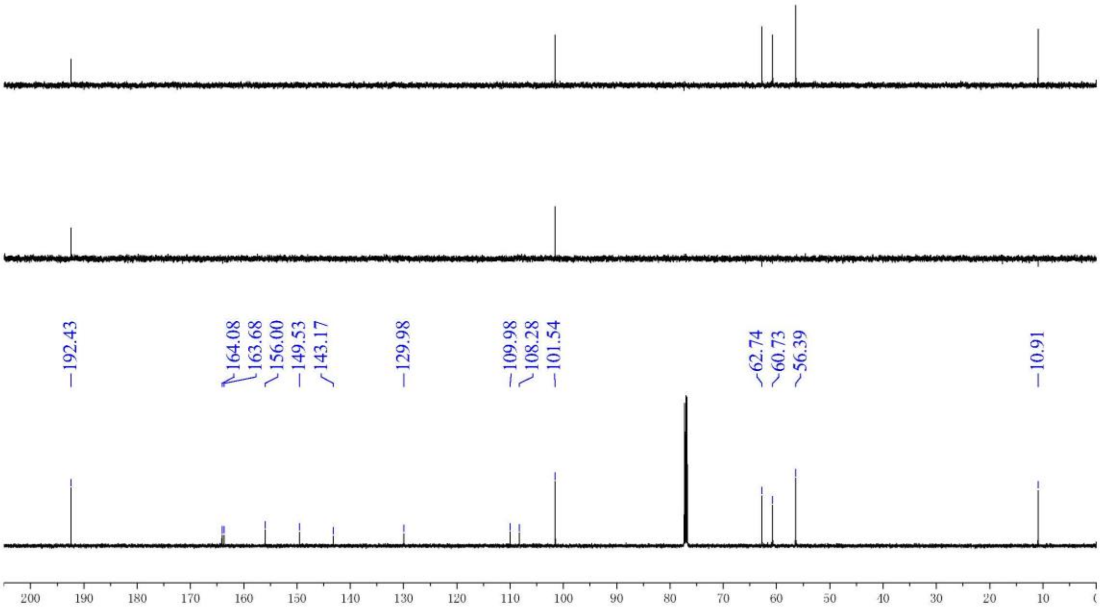

^{13}C-NMR spectrum of **6** recorded in $CDCl_3$ at 125MHz

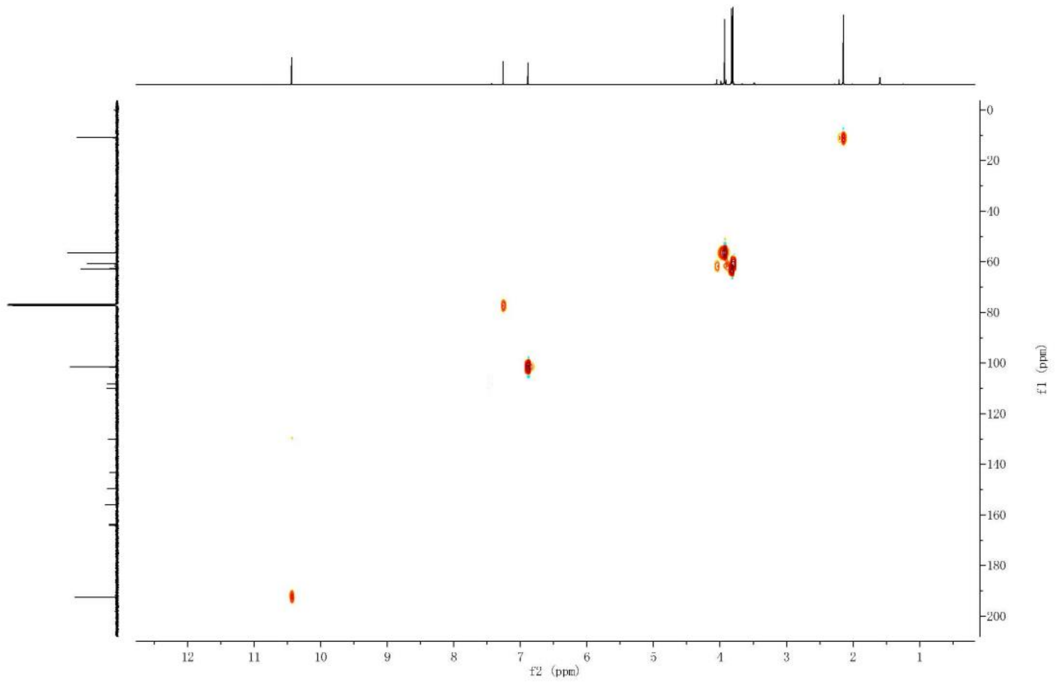

HSQC spectrum of **6** recorded in $CDCl_3$

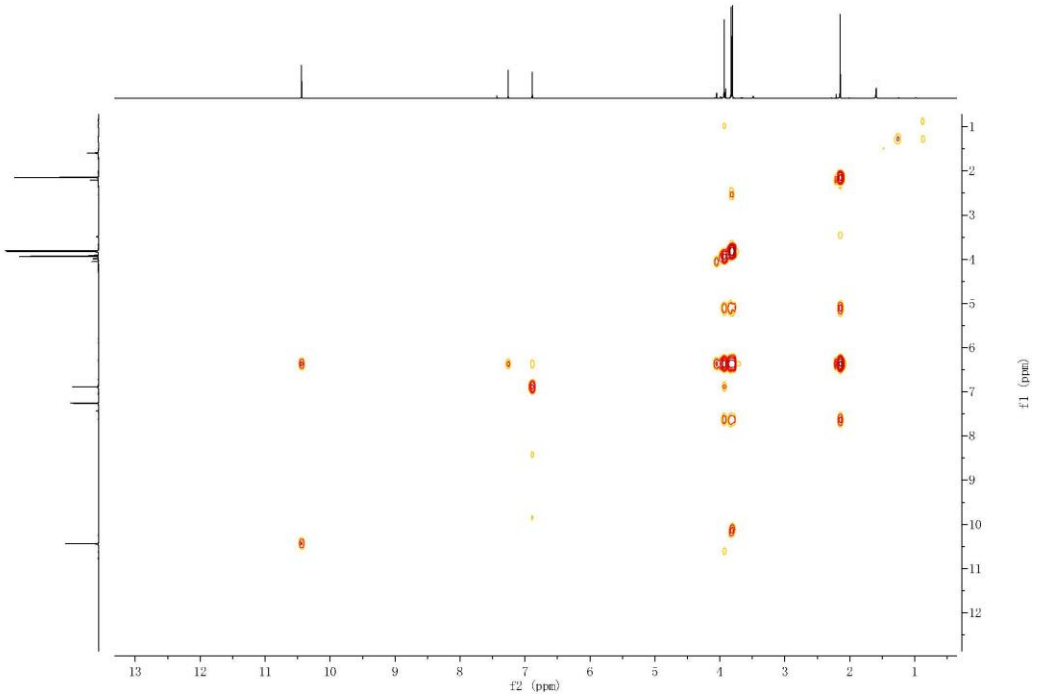

^{1}H-^{1}H COSY spectrum of **6** recorded in $CDCl_3$

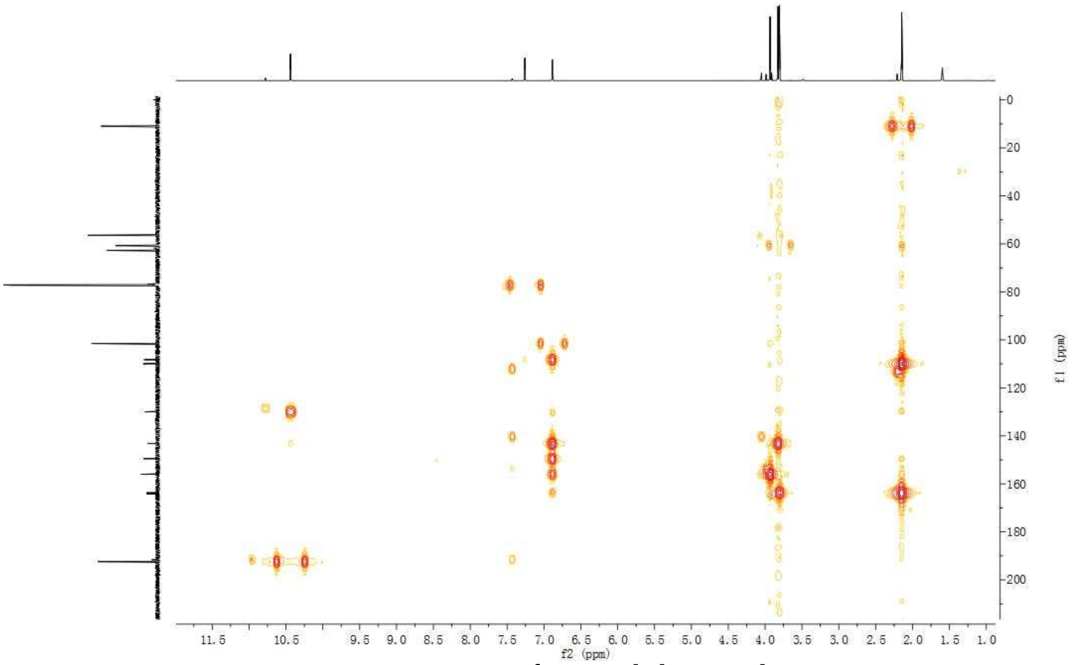

HMBC spectrum of **6** recorded in $CDCl_3$

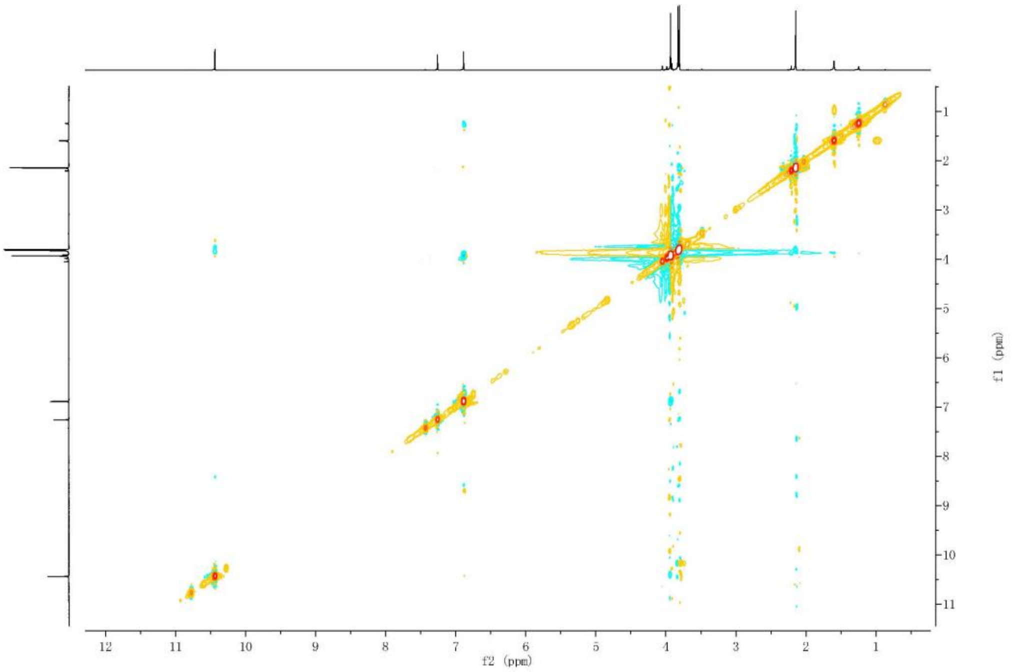

ROESY spectrum of **6** recorded in $CDCl_3$

Data File: E:\百度云同步盘\昆植所\实验室\LC-MS结果\先花铃子香\Cpx-A-6~7合并各部分\Cpx-A-6-3各部分\Cpx-A-6-3-1各部分\2017-07-21 LCMS...

Elmt	Val.	Min	Max	Elmt	Val.	Min	Max	Elmt	Val.	Min	Max	Use Adduct
H	1	0	300	O	2	0	50	Br	1	0	0	H
C	4	0	150	S	2	0	0					Na
N	3	0	10	Cl	1	0	0					

Error Margin (ppm):	50	DBE Range:	0.0 - 30.0	Electron Ions:	both
HC Ratio:	unlimited	Apply N Rule:	yes	Use MSn Info:	no
Max Isotopes:	all	Isotope RI (%):	1.00	Isotope Res:	10000
MSn Iso RI (%):	75.00	MSn Logic Mode:	AND	Max Results:	800

Event#: 1 MS(E+) Ret. Time : 8.467 Scan# : 1748

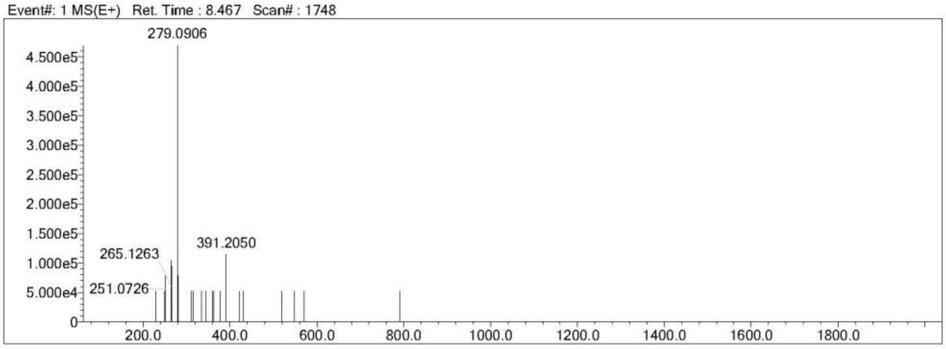

Measured region for 279.0906 m/z

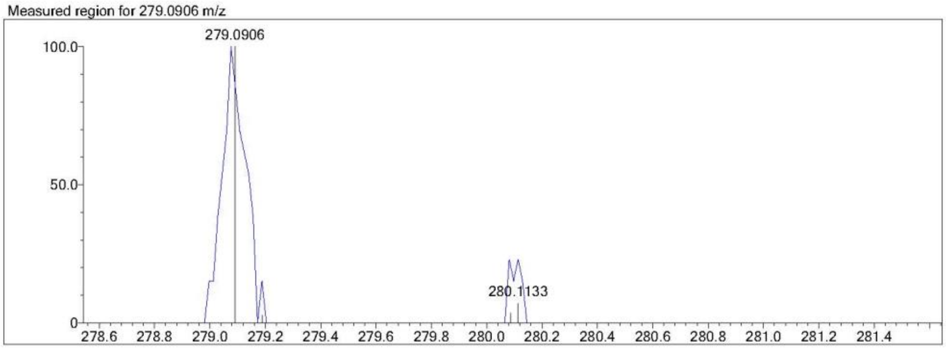

C14 H14 O6 [M+H]+ : Predicted region for 279.0863 m/z

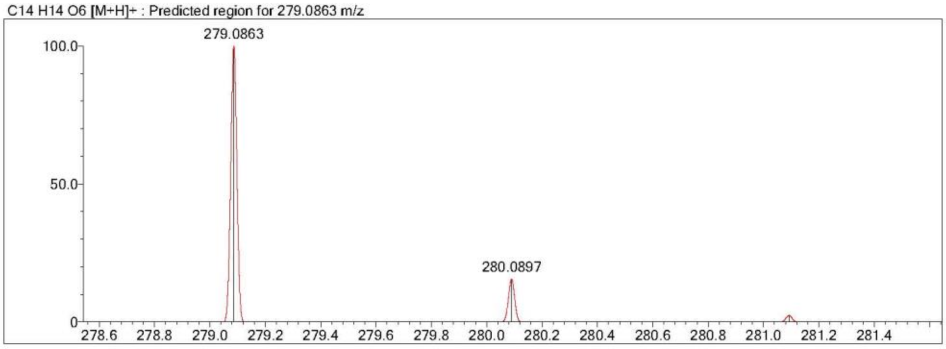

Rank	Score	Formula (M)	Ion	Meas. m/z	Pred. m/z	Df. (mDa)	Df. (ppm)	Iso	DBE
10	7.41	C14 H14 O6	[M+H]+	279.0906	279.0863	4.3	15.41	28.97	8.0

HR-ESI-MS spectrum of **6**

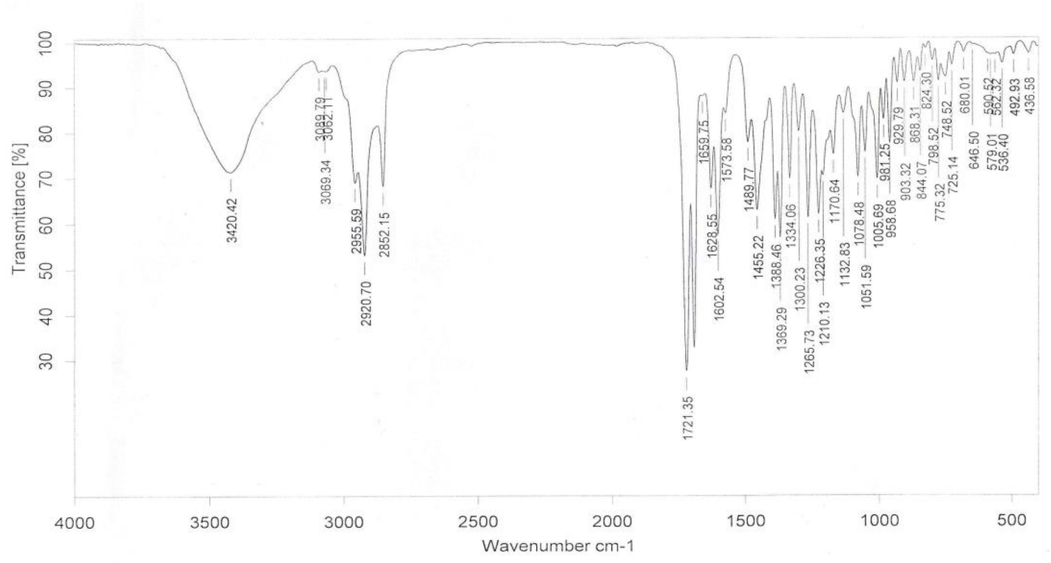

Sample : Jdzt-133	Frequency Range : 399.246 - 3996.32	Measured on : 26/09/2017	
Technique : KBr压片	Resolution : 4	Instrument : Tensor27	Sample Scans : 16
Customer : 170926IR11	Zerofilling : 2	Acquisition : Double Sided,For	

IR spectrum of **6**

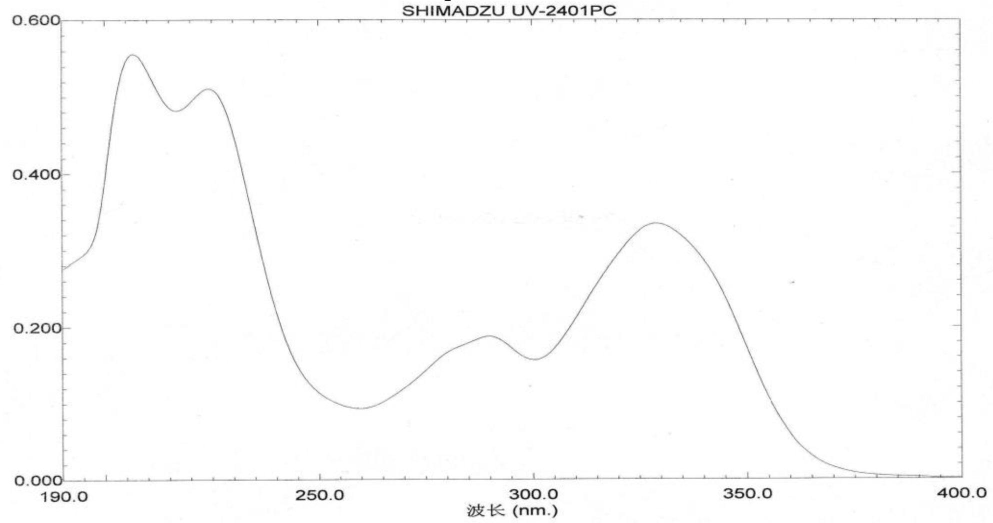

文件名: JDZT-133

JDZT-133

样品浓度：0.0331毫克/毫升
溶剂：甲醇

创建于: 10:14 17-11-20
数据: 原始

测量模式: Abs.
扫描速度: 中速
狭缝: 5.0
采样间隔: 0.2

否.	波长 (nm.)	Abs.
1	329.00	0.3344
2	290.00	0.1874
3	224.20	0.5104
4	206.60	0.5560

UV spectrum of **6**

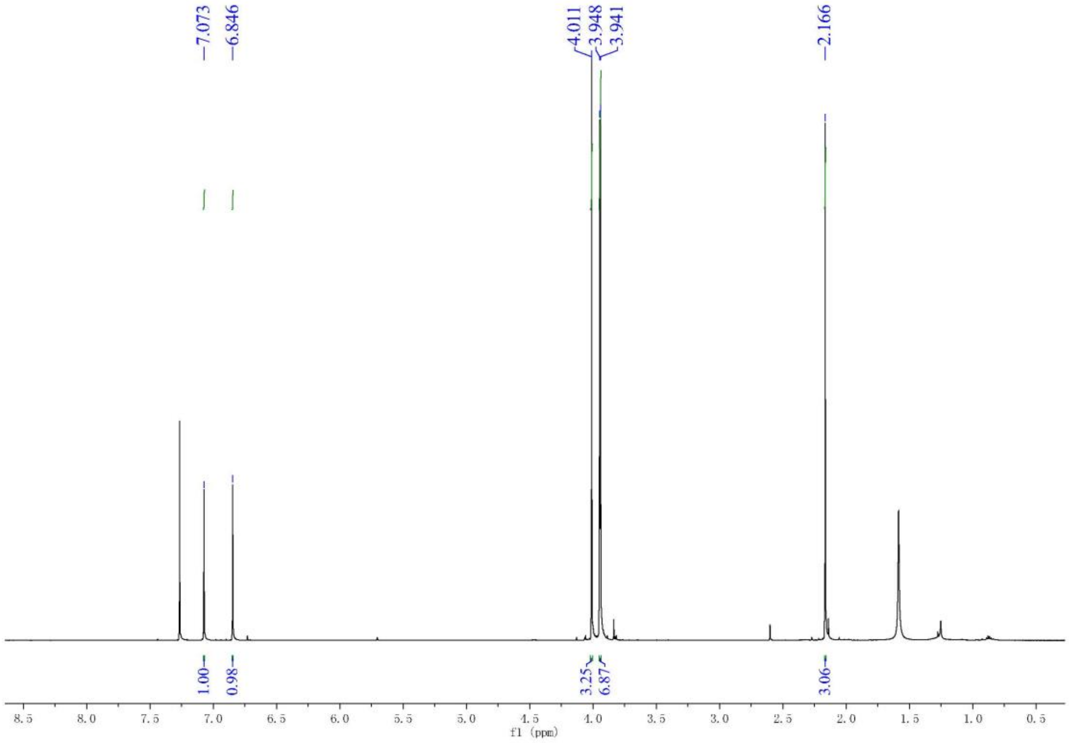

^{1}H-NMR spectrum of **7** recorded in $CDCl_3$ at 600MHz

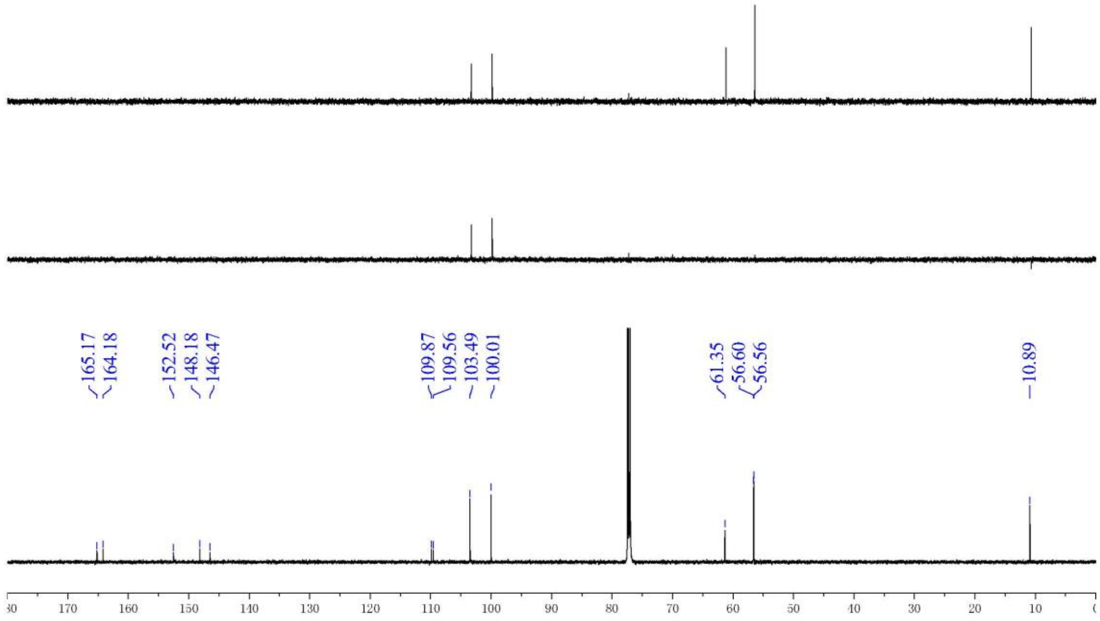

^{13}C-NMR spectrum of **7** recorded in $CDCl_3$ at 150MHz

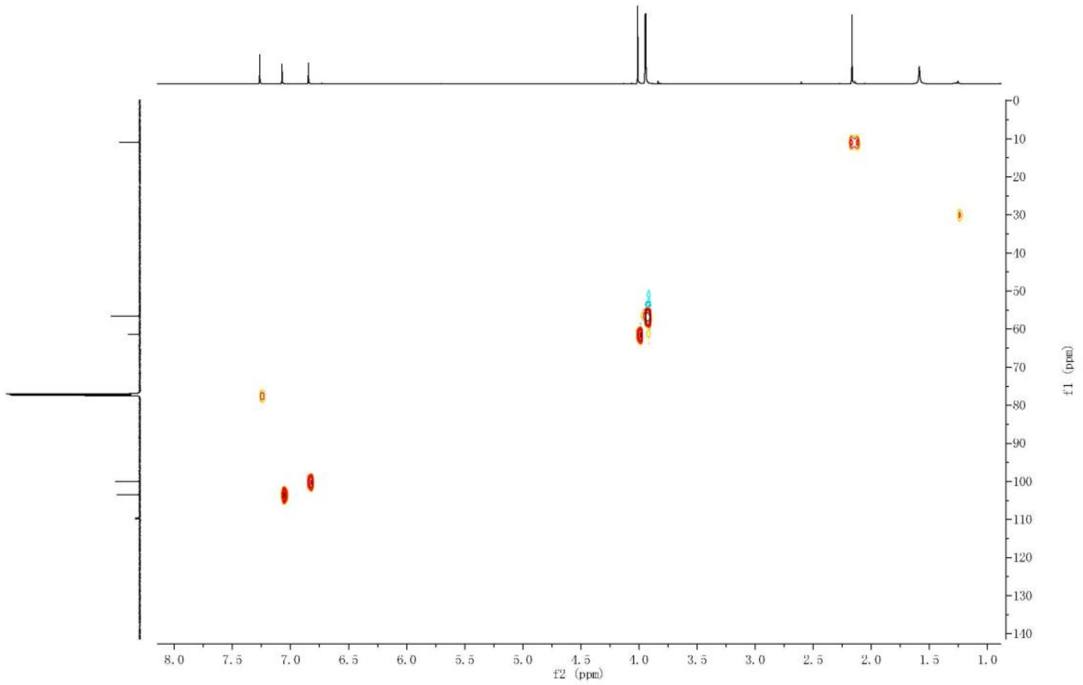

HSQC spectrum of **7** recorded in $CDCl_3$

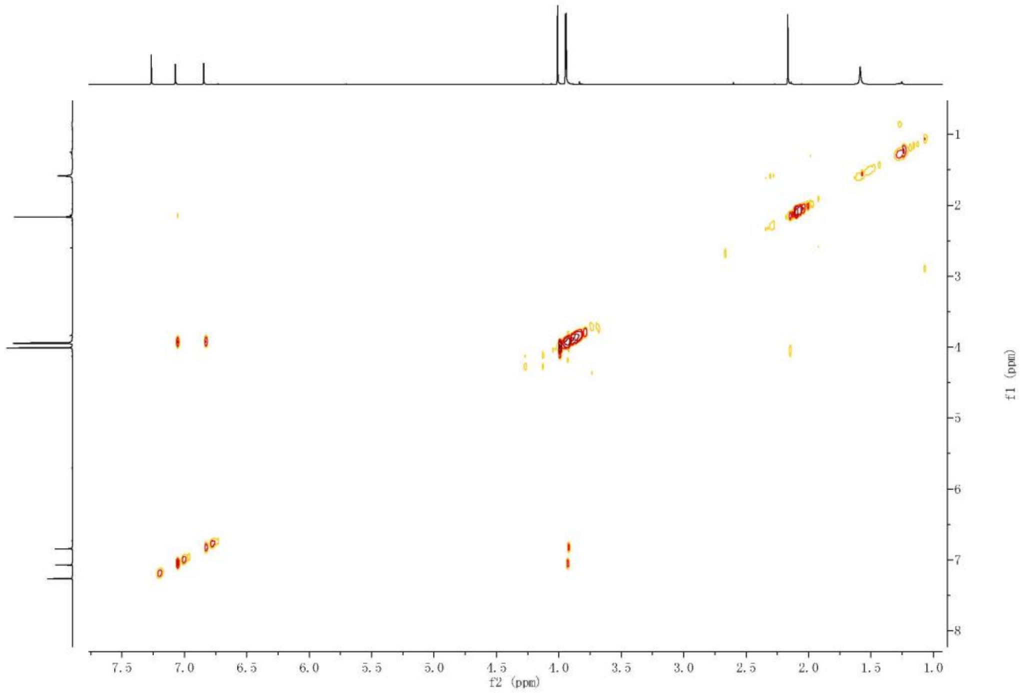

^1H-^1H COSY spectrum of **7** recorded in $CDCl_3$

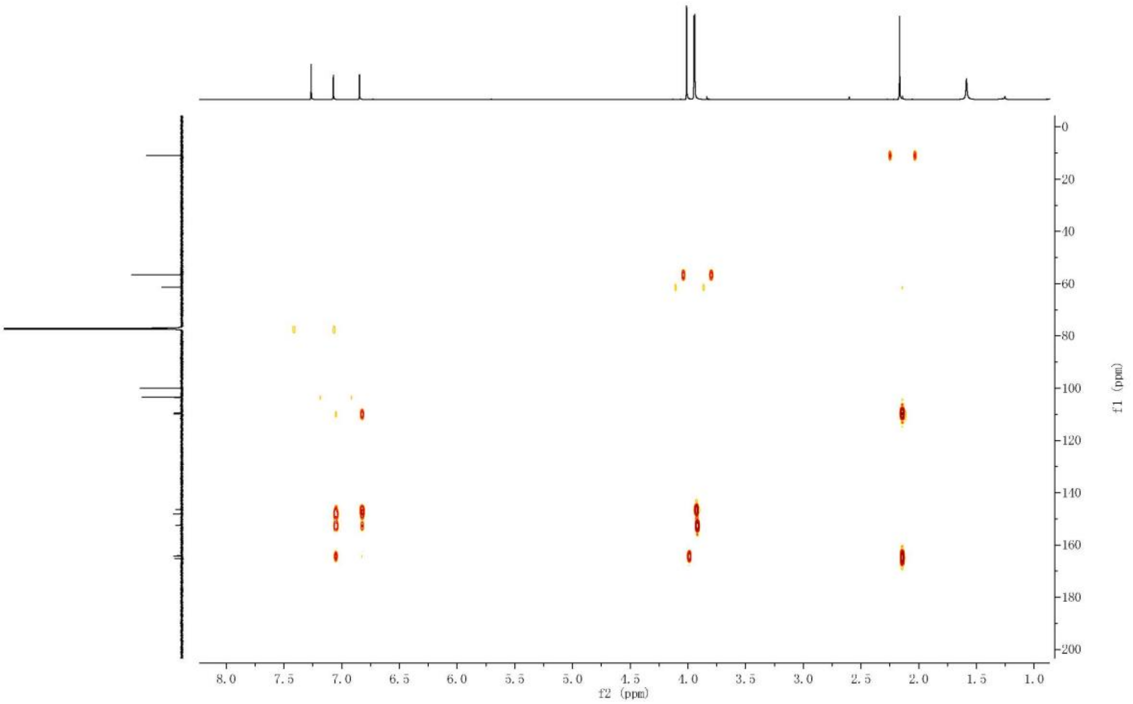

HMBC spectrum of **7** recorded in $CDCl_3$

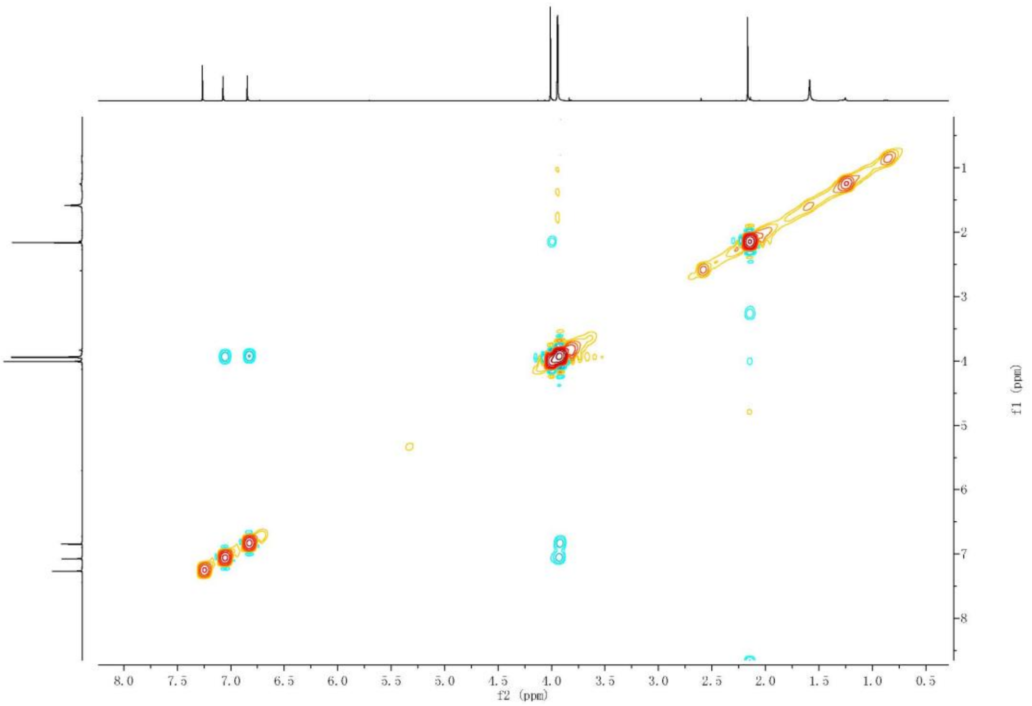

ROESY spectrum of **7** recorded in $CDCl_3$

Formula Predictor Report - Cpx-A-10-5-3-5_2017-06-07 LCMS_201767_16.lcd Page 1 of 1

Data File: E:\百度云同步盘\昆植所\实验室\LC-MS结果\先花铃子香\Cpx-A-10各部分\Cpx-A-10-5各部分\Cpx-A-10-5-3\Cpx-A-10-5-3-5_2017-06-07 ...

Elmt	Val.	Min	Max	Elmt	Val.	Min	Max	Elmt	Val.	Min	Max	Use Adduct
H	1	0	300	O	2	0	50	Br	1	0	0	H
C	4	0	150	S	2	0	0					Na
N	3	0	10	Cl	1	0	0					

Error Margin (ppm):	50	DBE Range:	0.0 - 30.0	Electron Ions:	both
HC Ratio:	unlimited	Apply N Rule:	yes	Use MSn Info:	no
Max Isotopes:	all	Isotope RI (%):	1.00	Isotope Res:	10000
MSn Iso RI (%):	75.00	MSn Logic Mode:	AND	Max Results:	800

Event#: 1 MS(E+) Ret. Time : 9.237 Scan# : 1851

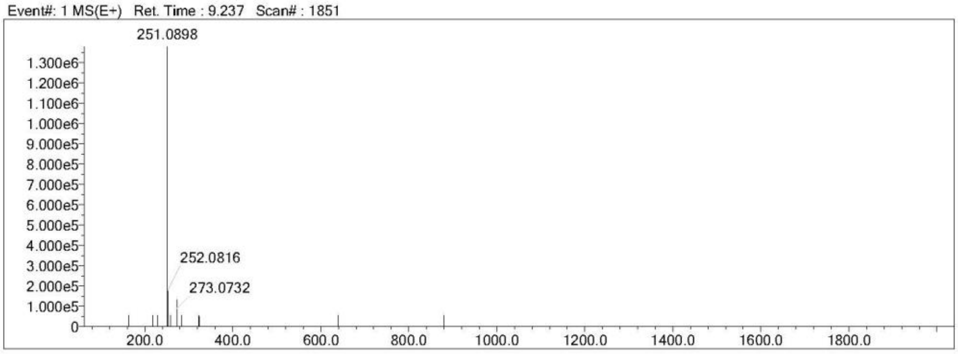

Measured region for 251.0898 m/z

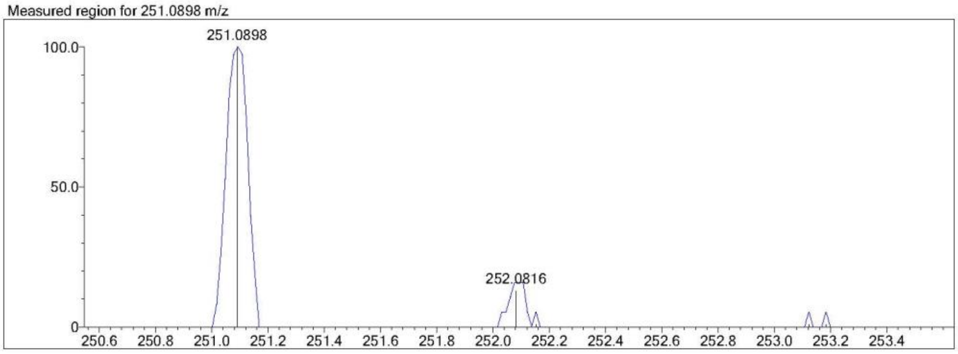

C13 H14 O5 [M+H]+ : Predicted region for 251.0914 m/z

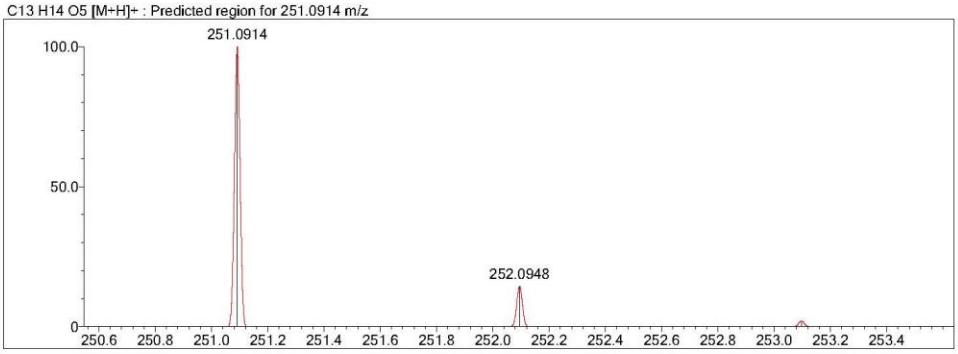

Rank	Score	Formula (M)	Ion	Meas. m/z	Pred. m/z	Df. (mDa)	Df. (ppm)	Iso	DBE
4	53.31	C13 H14 O5	[M+H]+	251.0898	251.0914	-1.6	-6.37	69.87	7.0

HR-ESI-MS spectrum of **7**

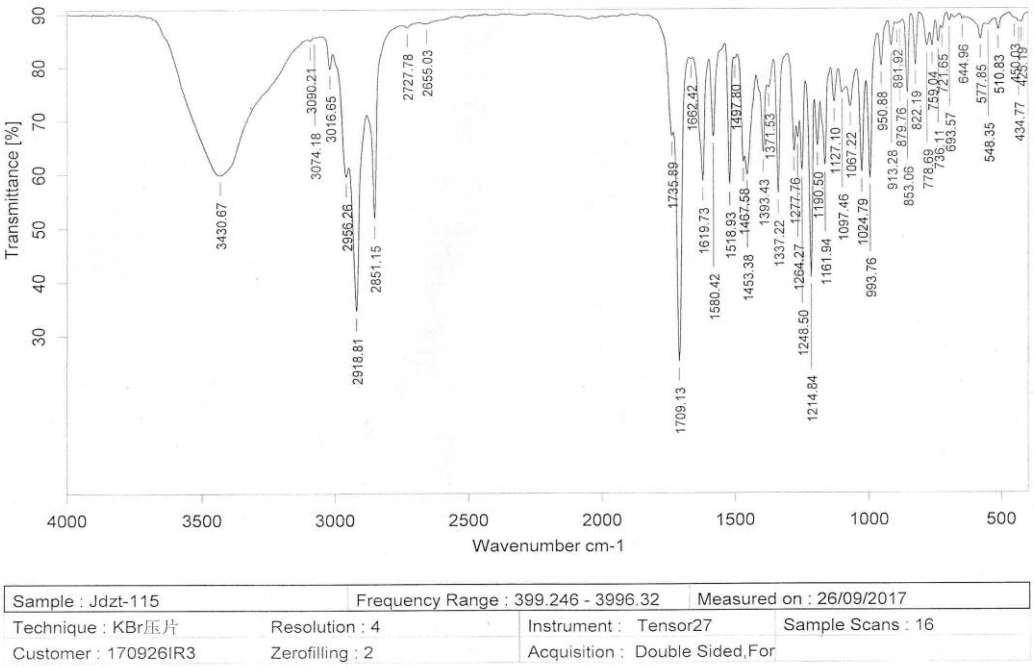

IR spectrum of **7**

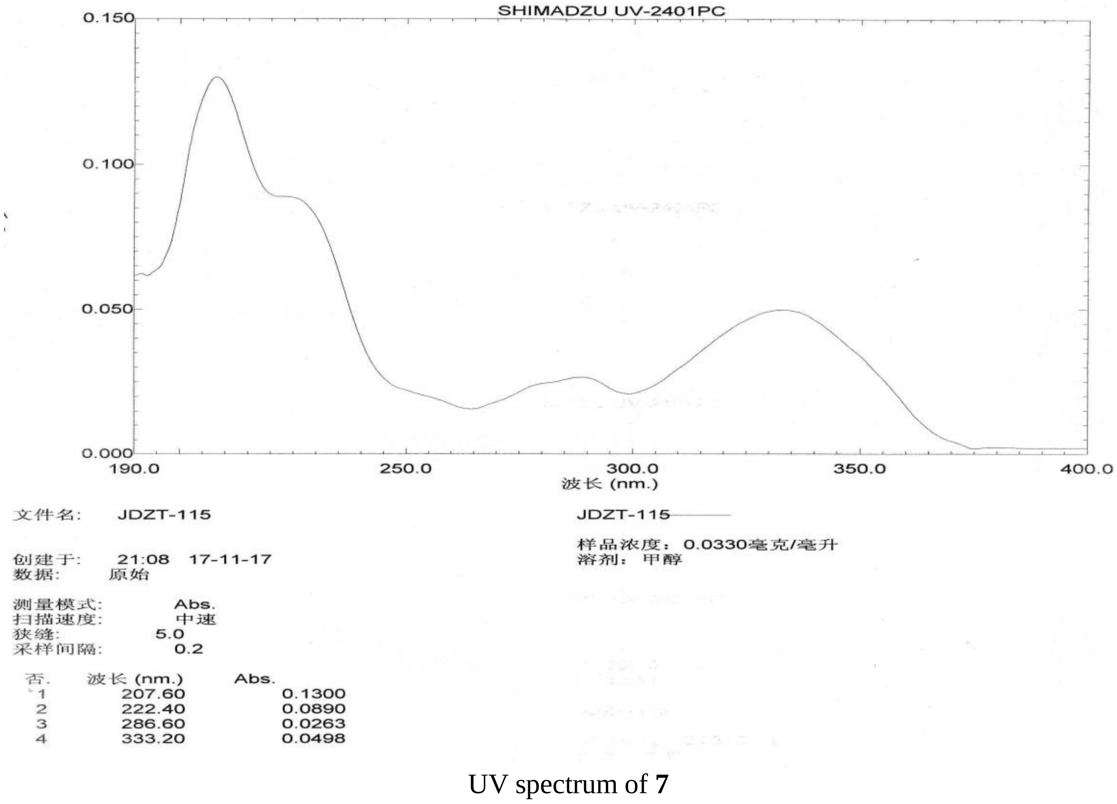

UV spectrum of **7**
